# Diurnal Nonlinear Recurrence Metrics of Skin Temperature and Their Association with Metabolic Hormones in Contrasting Climate Settings: A Randomized Cross-Over Trial

**DOI:** 10.3390/ijerph192215195

**Published:** 2022-11-17

**Authors:** Konstantinos C. Makris, Pantelis Charisiadis, Thibaut Delplancke, Nikolaos Efthymiou, Alessandro Giuliani

**Affiliations:** 1 Cyprus International Institute for Environmental and Public Health, Cyprus University of Technology, 3041 Limassol, Cyprus; 2 Environment and Health Department, Istituto Superiore di Sanità, Viale Regina Elena 299, 00161 Rome, Italy

**Keywords:** climate, sensors, recurrence, temporal, metabolism, synchronization

## Abstract

The urban overheating phenomenon in Mediterranean cities is a societal challenge with vast implications for the protection of public health. An additional analysis of the pilot TEMP randomized 2 × 2 cross-over trial was set up, using wearable sensor-based skin temperature measurements (n = 14). The study objectives were to: (i) assess the recurrence patterns of skin temperature measurements in individuals spending time in two climatologically contrasting settings (urban versus mountainous), and (ii) evaluate the association between the diurnal nonlinear recurrence quantification analysis (RQA) metrics and metabolic hormone levels. The intervention was a short-term stay (5–7 days) in a mountainous, climate-cooler setting (range 600–900 m altitude), which is about a 1 h drive from the main urban centres of Cyprus. The RQA analysis showed a blunting phenomenon on the nonlinear temporal dynamics of skin temperature time series observed in the urban setting. Compared with the mountainous setting, a more stable (and thus less adaptive) profile of skin temperature dynamics in the urban setting appeared, being less deterministic and with a smaller degree of complexity. No significant (*p* > 0.05) associations were observed between the leptin or cortisol and any of the skin temperature dynamical descriptors. However, there were marginal associations between the adiponectin and laminarity (beta = 0.24, 95%CI: −0.02, 0.50, *p* = 0.07) and with determinism (beta = 0.23, 95%CI: −0.037, 0.50, *p* = 0.09). We found dysregulations in skin temperature temporal dynamics of the study population while residing in the urban setting when compared with the cooler mountainous setting; these dysregulations took the form of reduced cycle duration and complexity, while skin temperature dynamics became less responsive to perturbations and less regular in magnitude. More research is needed to better understand heat stress temporal dynamics and their influence on human health. Trial registration: This trial is registered with ClinicalTrials.gov; number: NCT03625817.

## 1. Introduction

The climate and health nexus is continuously gaining momentum in a changing climate that adversely impacts planetary health [1]. A recent report by the World Health Organization (WHO) calls for the immediate adoption of interventions to reduce urban overheating [2]. Most scientific evidence has been collected on ambient outdoor temperature effects on human health [3,4]. Evidence on associations between indoor air temperatures and health outcomes is scarce. The literature suggests linkages between indoor air temperatures >26 °C and an increased proportion of respiratory distress calls (*p*  =  0.056) during summer in New York City [5].

Several studies have noted differences in environmental stressor exposure (e.g., particulate matter) between actual personal exposure measurements and estimates based on central monitoring station values [6]. This represents a historic issue in environmental epidemiology as the specific impact of exposure errors on effect estimates is not straightforward and depends on several factors, including study design, types of error, and the relationships between the outcome and the independent variables [7,8,9]. The growing availability and lower cost of wearable sensors allows for improving exposure measurement errors by providing individual-level measurements that are closer to the true personal exposure value. The choice of wearable sensors finds increasing use in environmental epidemiology studies, especially in those applying the human exposome framework and its exposomic tools, utilizing sensor data for multiple exposure stressors [10,11,12].

The deployment of such wearable sensors allows for capturing the exposure variance in 24-h skin temperature measurements as a result of human mobility and activity across different indoor and outdoor (micro)environments [10]. A diurnally varying temperature profiling was observed indoors for the majority of participants with a higher number of temperature peaks recorded indoors than outdoors [10]. Skin temperature is known to respond quite quickly to changes in ambient temperatures (within seconds) [13]. These time–activity varying patterns are often characterized by nonlinear temporal trends that are hard, if not impossible, to capture with standard biostatistical algorithms, such as linear regression models.

The body’s core temperature system is well controlled by the circadian clock and peripheral systems such as the thyroid gland. Skin temperature may be more responsive to episodic or short-term temperature gradient changes during the day in an ambient environment than the body core temperature system. As such, recurrence patterns of body or skin temperature as the body attempts to thermoregulate its core temperature and maintain homeostasis become highly important and they are characterized by dynamically evolving cyclic processes. It is important to stress that ensuring an interfacial synchronization of interacting systems will likely maintain a resilient biological state, whereas, on the contrary, systematic disruptions in synchronization (desynchronization) will likely lead to differential toxicity [14]. The global dimension of chronos (time in Greek) entails a dynamic equilibrium brought about by transient and temporally dynamic input systems or processes.

Synchronization or *synchronie* (in French), a term invented by the Swiss glossologist F. de Saussure, is defined as the totality of elements that operate during the same time period within a system, including the network of interactions between the system’s elements. Besides their entrainment with external cycles (e.g., the day/night circadian rhythm), organisms host a multiplicity of endogenous oscillatory temporal structures. This is an obliged consequence of the fact that any biological process encompasses a relation network among its players by being concurrently confined into a finite space [15]. From single protein molecules whose internal oscillatory dynamics stems from the mutual relationship among amino acid residues solicited by thermal noise [16] to ecological systems whose relative abundance of species oscillation is driven by their relative position in the food web [17], everything oscillates. However, such characteristic periods and amplitudes of natural oscillations span many orders of magnitude, and they can be modified by external environmental stressors. These modifications can alter the harmonic character of the oscillatory behaviour and disrupt their stationary character [18]. This requires the adoption of non-linear time series analysis tools [19] in order for the temporal dynamics of disease processes to be captured and appreciated at the right scale.

Typical examples of such dynamically oscillating proportional and reversible disease models [20] would be the effects of proportional dosing effects of pesticides on their rapid binding to cholinesterase enzyme, blocking the enzyme and causing a series of respiratory, gastrointestinal or central nervous system symptoms due to accumulation of acetylcholine in red blood cells; another example would be the severity of respiratory symptoms (cough, phlegm) being proportional to use of cigarettes packs per day, or the irritant respiratory effects associated with proportional ground level ozone exposures. For example, exposures to a chemical that do not saturate the enzymatic capacity either by being low in concentration or because exposures occur outside a specific chrono-window of a day with higher enzymatic activity would result in larger effect size per unit of exposure than other cases.

The above-mentioned examples of environment–health associations have never been studied before under the proposed temporal dynamics mode of finer temporal resolution for monitoring and analysing time series for both exposures and effects [14,18]. It has been shown earlier that studying the temporally dynamic network of systems would facilitate the detection/capture of critical transitions of early-warning signals of the pre-disease state so as to prevent drastic deterioration [15]. The transition from a resilient to a perturbed state cannot be captured with the conventional quantitative tools of environmental epidemiology for the aforementioned reasons. The temporal dynamics towards a perturbed state involves a myriad of steps that cannot be known in detail, calling for a global search for ‘network biomarkers’ that are sensitive and responsive to changes in correlation degree more than to actual values of single players [15].

The need for a deeper analysis of the daily patterns of skin temperature fluctuations emerged from the findings of the main analysis of the TEMP trial [10,11]. The main TEMP analysis showed alterations in metabolic hormones (e.g., leptin) associated with a short-term stay (5–7 days) in climatologically cooler areas than those observed in geographically adjacent urban settings (~1-h driving distance) during a typical Mediterranean summer [10,11]. Towards this, an additional analysis of the pilot TEMP study dataset was set up, using the finely resolved time series data that were available, based on the wearable sensor-based skin temperature measurements. Thus, the study objectives were to: (i) assess the recurrence patterns of skin temperature measurements in individuals spending time in two climatologically contrasting settings (urban versus mountainous), and (ii) evaluate the association between the recurrence quantification analysis (RQA) metrics and metabolic hormone levels.

## 2. Methods

### 2.1. Trial Oversight

This study involved participants from the TEMP 2 × 2 cross-over pilot trial of healthy, non-obese adults (n = 41) (NCT03625817). Participants were included in this analysis, if they had complete skin (excluded if ≥80% skin temperature values were below 30 °C during the 24-h) or personal air temperature measurements in both settings (n =16). Detailed information about the study methodology can be found in the original publication [11]. In brief, the trial studied the influence of setting and skin temperature or personal air temperature gradient on metabolic hormone profiling. Data and sample collection took place in Cyprus from July until the end of September 2018. 

No participants were involved in setting the research question or the outcome measures, nor were they involved in developing plans for the design or implementation of the study.

The trial protocol was approved by the Cyprus National Bioethics Committee (ΕΕΒΚ/ΕΠ/2018/33) and written informed consent was signed by the participants. The trial was performed in accordance with the principles of the Declaration of Helsinki. The authors assume responsibility for the accuracy and completeness of the data and analyses, as well as for the fidelity of the trial.

### 2.2. Trial Population and Intervention Characteristics

Eligible participants were healthy adults between 20–60 years old, having their permanent residence in one of the two largest cities of Cyprus (Nicosia and Limassol), and who intended on staying in the Troodos area (a mountainous rural area in Cyprus) for a period of at least 5 to 7 consecutive days between July–September 2018. Recruitment was made possible via the dissemination of flyers, radio ads and telephone communication with governmental and other non-profit organizations (Troodos Development Company, Troodos Tourist Board, etc.) that contacted community leaders of the mountainous area and helped us to collect and create a list of potential participants. Using this list, potential participants were informed about the study, screened for eligibility criteria and agreed to participate via telephone communication. Exclusion criteria were applied to those who were pregnant, obese, suffering from a chronic condition (hypertension, diabetes, metabolic syndrome, cancer), receiving pharmaceutical treatment for impaired glucose levels, hypertension, antidepressants or thyroxin medication for thyroid disorders, or those who had travelled to another country in the week prior to the study’s initiation. The intervention was a short-term (5–7 days) stay in the cooler-climate rural areas of the Troodos mountains.

### 2.3. Randomization and Masking

Random allocation of the intervention was carried out in blocks to two groups that differed in the sequence of the treatments. Block randomisation was performed using the function RAND() in Excel with a randomly generated list by a research investigator with no trial involvement. Group 1 included volunteers initially staying in their permanent house in the urban area and then moving to the rural/mountainous area for at least 5–7 days; Group 2 included individuals who initially stayed in the mountainous area for at least 5–7 days and then returned to their permanent residence in the urban area.

The blinding of the participants to group assignment was not possible with the present study design. The blinding of the researchers to the subjects’ identity was achieved by coding all study materials (urine containers, questionnaires, and diaries). The study personnel were not informed of the group assignment.

### 2.4. Trial Procedures

Participants spent their short-term stay in the rural communities of the mountainous Troodos area in Cyprus with an altitude range of 600–900 m above sea level.

The researcher visited the participant one day before the first sampling day (4–6 days after being in the allocated setting). Upon giving informed consent, the participant completed the baseline questionnaire and the Munich ChronoType Questionnaire. Study materials were provided. Participants were asked to collect all urine voids separately, to note their day’s activities in the activity diary and to wear the temperature sensors continuously until mid-day (noon) of the day following the sampling day. A washout period of at least 9 days between settings was observed (average: 22 days, range: 9–41 days).

### 2.5. Sensors

Wearable sensors of skin and personal air temperature, including activity tracking (as a surrogate marker of physical activity) (e-TACT, BodyCAP Medical, Hérouville-Saint-Clair, France) were used. The skin sensor was attached to the armpit, while the personal air temperature sensor was attached as a hanging tag on the individual’s chest. For the skin temperature sensor, only data collected with temperatures ≥ 30 °C were included in the final data analysis (n = 14), while for personal air sensor data we had 15 eligible participants. The following input parameters were set for the sensors: temperature measurement period (1 min), sampling frequency (50 Hz), the actimetry period (1 min), the accelerometer sensitivity threshold to consider an activity as important or not (0.1 g) and the measurement range (2G). The activity sensor tracking mode used the classic TAT method (time above threshold).

### 2.6. Urine Sample Collection

Two 24 h periods of urine sampling per participant were obtained, a 24 h sampling period at the end of the 5–7 days period in the urban setting and another 24 h sampling period at the end of the mountainous/rural setting period. On the sampling day, the first morning void urine sample was discarded and the first morning void of the following day was obtained. All bladder content was emptied into separate vials whenever participants urinated. The time stamp of each urination and its volume were recorded in a diary. All samples were stored in the freezer until handed to the laboratory, where aliquoting was performed and samples were stored at −80 °C until analysis.

### 2.7. RQA Analysis

The notion of recurrence is simple: for any ordered time series, a recurrence is a value which repeats itself within an assigned tolerance (=radius). Thus, given a reference point, X0, and a sphere of radius r centred on it, a point X is said to recur if:

Br(X0) = {X:||X − X0|| ≤ r} (1)

Equation (1) can be generalized in terms of the distance between any two i, j points considered in a Euclidean space: a recurrence is scored anytime the distance between two i, j points is lower than a pre-determined threshold r.

In the case of a time series of length n, the n × (n − 1)/2 distinct pairwise distances between the n points are computed in terms of Euclidean distances in a phase space having as axes p lagged copies of the series, with *p* = embedding dimension.

If we let *p* = 3, out of an original series S = 10, 11, 21, 32, 41, 35, 40, 19, then by shifting the series by a fixed lag, we obtain the corresponding 3-dimensional phase space:

10  11  21

11  21  32

21  32  41

32  41  35

41  35  40

35  40  19

40  19  .

19  .     .

The above embedding matrix (EM) has as rows subsequent epochs of the series of length p. The matrix of the Euclidean distances between all the rows (epochs) of EM is converted into a binary matrix, recording a value of 1 (scoring of a recurrence) every time the distance is lower than r, and 0 otherwise.

**Figure 1:**
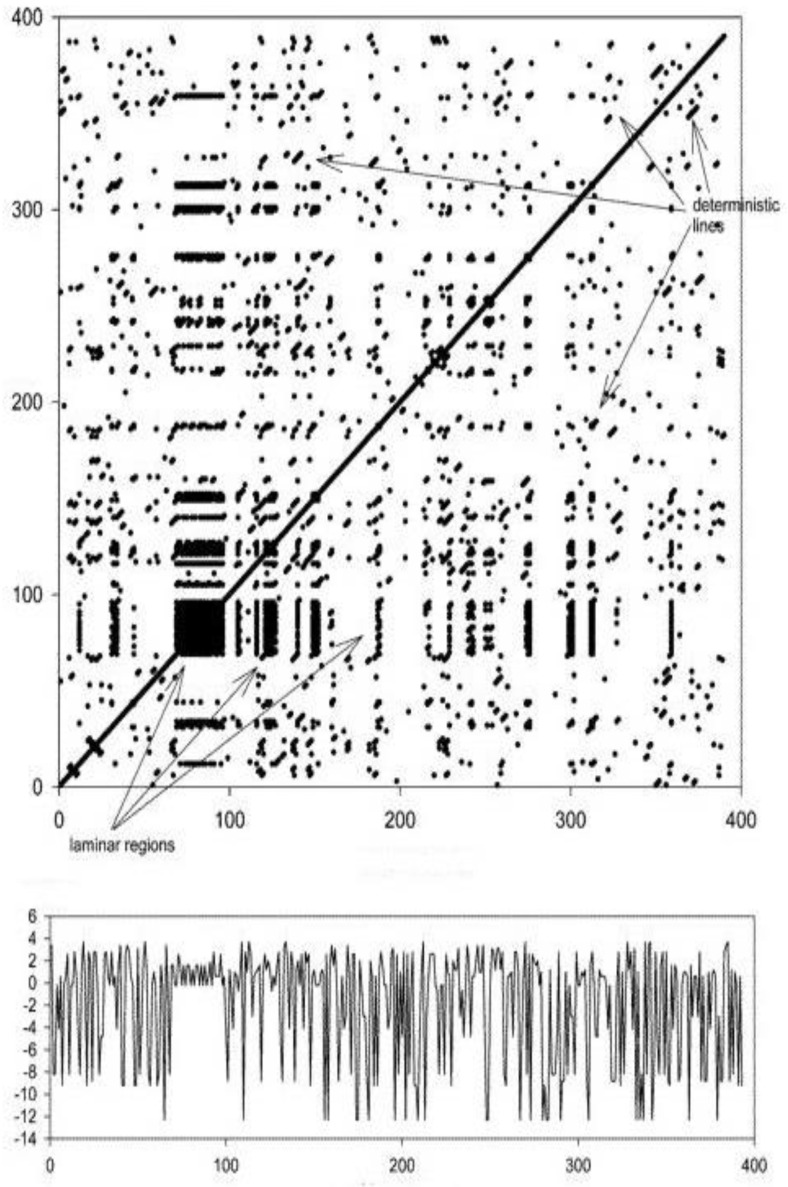
The black dots correspond to recurrences of pairs of points having their time coordinates reported on the x and y axes of the graph. Adjacent-in-time recurrences define deterministic lines (parallel to the main diagonal), while laminar regions are horizontal (vertical) lines of recurrences in the plot. Both deterministic and laminar lines are indicated by arrows in the figure. In this case, embedding dimension *p* = 4, and the radius was set to 1/10 of mean distance (modified from [21]).

Such a binary distance matrix is called a Recurrence Plot (RP) and is graphically depicted by a black dot at every recurrent pair. Given the symmetric character of distance, RP aspect is symmetrical along the main diagonal. We report here an example of a recurrence plot (top panel), together with the original signal from which it was derived (Figure 1).

The RPs generate quantitative descriptors of the time series by different indexes describing the number and pattern of recurrent point; the simplest descriptor is recurrence rate (REC):

REC = 1/N^2^ SUM (Rij), being R = 1 if i, j recur, and 0 otherwise; N = time series length.

Determinism (DET) corresponds to the proportion of recurrences (Rij) appearing in sequence, with respect to the total number of recurrences:

DET = SUM(Drij)/SUM(Rij), where Drij are those recurrences forming diagonal lines.

Laminarity (LAM) is the analogue of DET as for horizontal (vertical) lines, namely is the proportion of recurrences appearing in horizontal (vertical) lines:

LAM = SUM(Lrij)/SUM(Rij) where Lrij are those recurrences forming horizontal(vertical) lines.

The above three RQA metrics can be considered as the basic ones that in turn generate secondary indexes, namely:

Entropy (ENT) corresponds to the Shannon Entropy of the length distribution of deterministic lines. Given the definition of deterministic lines as recurrences appearing in sequential order, they can form lines with a length going from 2 to N; the relative proportion of these length classes gives rise to ENT expressed in terms of the Shannon entropy index as:

ENT = SUM (p(l)log(p(l)), where the SUM is extended from 2 to N and p(l) = proportion of deterministic lines of length = l.

Divergence (DIV) corresponds to the inverse of MAXLINE (the maximum length of deterministic lines):

DIV = 1/MAXLINE.

The name divergence stems from the mathematical demonstration of the strong link between DIV and the first Lyapunov exponent of a dynamical system [22] that corresponds to the ‘instability’ of the dynamics. Analogously, Lmean is the average length of deterministic line and Vmean the average length value of vertical (laminar) lines.

The slope of the linear relation linking the number of recurrences (Rij) and the distance from main diagonal of RP is called TREND (TRE). This is a measure of non-stationarity of the series: keeping in mind the meaning of recurrences, an RP having recurrences only in the vicinity of main diagonal implies a drift affecting the series that changes its statistics along the time dimension (i.e., progressively going away from the main diagonal of RP).

The brief discussion of the meaning of TRE allows us to understand the huge success RQA enjoys in fields as diverse as theoretical physics and psychology to ecology, molecular biology and engineering [21,22,23,24]. RQA does not impose any constraint of length, stationarity, functional form, type of dynamics, while allowing for an immediate appreciation of the essential features of the studied dynamics. Thus, an increase in determinism points to the approaching of a phase transition (e.g., an epileptic seizure in the case of Electroencephalography, the onset of fatigue in electromyography) or a more ‘constrained’ and less ‘environmentally sensible’ dynamics [25,26,27,28]. On the other hand, an increase in ENT implies a richer deterministic dynamics profile with the activation of a higher number of drivers.

In summary, the following recurrence quantification analysis (RQA) metrics were calculated, serving as relevant nonlinear markers of changes in dynamical systems and physiological states [29,30,31]: (i) recurrence rate (REC) denoting the density of recurrence points in a recurrence plot; (ii) determinism (DET), which is the percentage of recurrence points that form diagonal lines in the recurrence plot of minimal length; (iii) laminarity (LAM), which is the number of recurrence points forming vertical lines (intermittency); (iv) divergence (DIV), which equals the inverse of Lmax, where Lmax is the maximal diagonal line length; (v) entropy (ENT) denoting the complexity of the deterministic structure in the system; vi) trend (TRE), which is the regression coefficient of a linear relationship between the density of recurrence points in a line parallel to the LOI (line of identity) and its distance to the LOI, (vii) the mean diagonal line length (Lmean); and （viii) the mean vertical line length (Vmean).

The input parameters of the RQA analysis were: the embedding dimension (ED = 2), the number of lagged (time lag = 1) replicas of the original sampled series of temperature consecutive values, used to construct the Takens’ vectors and the maximum distance between two phase-space points (vector points having a number of components equal to ED) to be considered a recurrence, which was set as radius = 0.1 times the mean distance between distinct time point pairs. This threshold was selected to keep low REC and have a consistently high DET not affected by adjacent recurrences due to chance. This way, the saturation of recurrence is avoided (too many recurrent points can give rise to false deterministic lines). The time series of the skin temperature fluctuating dynamics were taken for the same subject sampled in both urban and mountainous environments considered as proxies of ‘business as usual’ (urban) and ‘more relaxed’ (mountainous) settings. The fact that RQA catches the features of the single-subject physiological dynamics [21] asks for a paired statistical analysis (paired *t*-test) for RQA descriptors comparing the two conditions on a single subject basis. This means that the observation of largely sign-invariant changes of RQA descriptors going from urban to mountain conditions is much more relevant than the average change over the entire population.

The minimal length of vertical/diagonal lines to be considered in the RQA plot were lmin = 2, while distance-to-border points near the border of the recurrence matrix were ignored when computing the RQA metrics (distance to border = 2). The non-linear RQA modelling and its metrics were constructed with the aid of the *nonlinearTseries* R package and the *ggplot* R package for visualization of RP using our custom code.

### 2.8. Outcomes

The primary outcomes of this study were the metabolic (leptin and adiponectin) and stress hormones (cortisol). Metabolic hormones (leptin and adiponectin) were quantified using validated kits for human urine (immunoassays, Elabscience, Houston, TX, USA). Cortisol was measured using gas chromatography coupled with mass spectrometry [32]. Urinary creatinine was determined using the picric acid-based spectrophotometric method (Jaffe method) [33].

### 2.9. Statistical Analysis

The baseline characteristics were presented overall and by study group. Categorical variables were described with sample size and percentages and compared by the chi-square test. Approximately normally distributed continuous variables were described with means and standard deviations (SD) and compared using the *t*-test. Non-normal continuous variables were described with medians and interquartile ranges (IQR) and compared using the Wilcoxon test. Variables with skewed distributions were log transformed to approximate normality. Biomarker values below the LOD were imputed to LOD/2. All outcome variables were adjusted for creatinine levels to account for urine dilution. The percentiles of each RQA metric were calculated overall and by setting, while log transformed RQA metrics by setting were compared using a paired t test comparing the subject recurrence dynamics in the urban and mountain settings. A principal component analysis (PCA) of the RQA metrics allowed for the calculation of the eigen values and associated variance for each principal component; we retained principal component 1 (PC1) for inclusion in the below mixed effect models.

For the analysis of the primary outcomes, mixed effect models were fitted for the RQA metrics (log-transformed) as a function of the setting (mountainous vs. urban) adjusted for age and sex and accounting for both between and within subject variability. Linear mixed effect models were also fitted for the adipokines and cortisol hormones (creatinine-adjusted and log-transformed) as a function of the RQA metric and the time (first morning vs. night) of the diurnal biomarker measurements (sample type), accounting for both between and within subject variability. All mixed effect models included participant-level (repeated measures within person) random intercepts with an unstructured covariance matrix.

One set of sensitivity analysis was undertaken using a radius of 0.01 to produce the recurrence plots. Statistical tests and 95% confidence intervals were two-sided with the statistical significance level set at 5%. All analyses were performed in R (v.5.2) with Rstudio (v.1.1.463).

## 3. Results

### 3.1. Participant Characteristics

From the initial pool of 41 volunteers who agreed to participate, a total of 31 volunteers were included in the original analysis [11], while a total of 16 volunteers were retained for this study, because these were the participants with fully available skin temperature measurements in both settings, minus two participants; one was excluded because ≥80% skin temperature values were below 30 °C, and another was excluded, because of abnormally high leptin levels (mean 3926 ± 209 ng/L) (n = 14). Most participants were females (78.6%) with a mean age of 42 years and normal BMI (Table 1). A high level of education was reported for the participants, with the majority holding at least a university/college degree (64%). At baseline, most participants were non-smokers (79%) with intermediate chronotype (50%), while about half of them reported that they never/rarely consume alcohol (57%) and reported spending about 5.4 h per day on screens (Table 1).

### 3.2. Nonlinear Temporal Temperature Dynamics and Their Metrics

**Figure 2:**
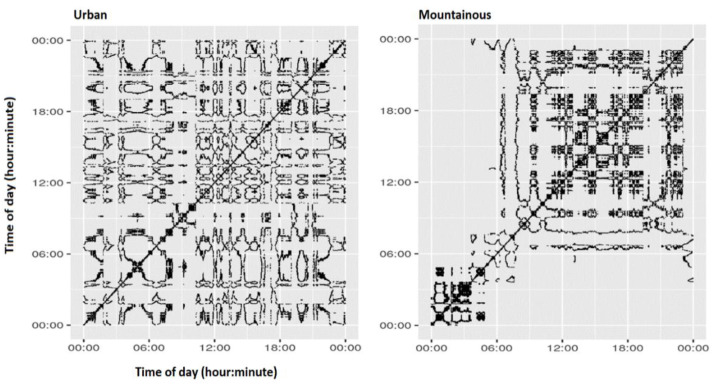
Indicative individual-level recurrence plots (RP) (24-h, 1-min intervals) for skin temperature gradients of the TEMP RCT with subjects spending time in an urban (left) setting and crossing to a cooler mountainous setting (right) during summer. The axes of the RPs correspond to subsequent time points, while darkened pixels mark recurrences, i.e., the dynamics returning to an already-visited state corresponding to the quasi-equivalence of the temperature value of the X and Y coordinates.

The individual-level recurrence plots (RP) based on the continuous 24-h measurement period using 1-min intervals for the skin temperature gradient are shown in Figure 2, from which all RQA metrics were calculated.

The average max daily air temperatures recorded using governmental station measurements followed the pattern of urban > mountainous settings, e.g., Nicosia (35 °C) > Limassol (33 °C) > Troodos (25 °C). Median values by setting for all RQA metrics can be found in the Supplementary File. The skin temperature profiling showed a diurnal pattern, with the highest values during the later afternoon towards the night and the lowest in the morning, which is consistent with earlier findings on distal and proximal skin temperature profiling regardless of the season [34]. Because of the diurnal character of the skin temperature measurements, the nonlinear temporal dynamics RQA algorithm was applied. Keeping in mind that DET corresponds to the proportion of recurrent pairs that occur along diagonal lines (i.e., appearing one after the other) out of total recurrent pairs, the low value of REC (overall mean REC = 0.06; number of recurrent pairs/total number of pairs) ensured that the observed deterministic line of high repetitiveness (mean DET = 0.91) did not occur by chance. The high DET value was indicative of a time series with a highly ordered structure (low system randomness). The high Lmean indicator (overall mean = 6.7) suggested the system’s stability in its attractor for a long time. The Lmax descriptor corresponds to the length of the longest deterministic line, with the DIV value (inverse of Lmax) being quite low (DIV = 0.005); this metric has been correlated with the so called ‘Lyapunov exponent’, i.e., the speed of change of a system’s dynamics [25]. The indicator of ENT denotes the degree of ‘richness’ of a deterministic structure. This parameter corresponds to the Shannon entropy of the length of the various deterministic lines in the RP. Therefore, if all diagonal lines have the same length, then ENT goes to zero; if there are many different lengths with almost equal frequency, ENT goes to the maximum.

The presence of several laminar states is indicative of intermittent patterns, suggesting the classical route to chaos; this was demonstrated in this study with the ‘ellipsoid’ figures (‘butterfly’ like patterns, Figure 1), pointing to ‘Brownian motion’ trajectories, i.e., to trajectories in which the system moves along nearby states and then returns to its original state. The Vmean descriptor corresponds to the length of the mean vertical line (Vmean = 7.92); the higher this is, the more unstable the system, with states changing more frequently. Long vertical lines (Vmean) suggest high laminarity (LAM = 0.94), i.e., intermittency-based patterns in the temporal dynamics of the skin temperature system. TRE was almost close to zero (TRE = −0.00005), suggesting the stationarity of the time series, i.e., the underlying system works with the same dynamics all along the observed time window; the more negative the TRE, the less stationary the system is.

The RQA metrics, when analysed by setting (urban vs. mountainous) showed interesting patterns. In effect, most of the monitored RQA metric values, i.e., Lmean, Vmean, DET, PC1, LAM, ENT and REC, were consistently higher (*p* < 0.05) in the mountainous setting than those observed in the urban setting (Table 2).

Thus, a blunting phenomenon on the nonlinear temporal dynamics of skin temperature time series was observed in the urban setting. Compared with the mountainous setting, a more stable (and thus less adaptive) profile of skin temperature dynamics in the urban setting appeared, being less deterministic and with a smaller degree of complexity. For the personal air temperature dataset, only the TRE, ENT, Vmean and Lmean metrics was significantly higher in the mountainous setting, while the rest of them were not (*p* > 0.05) (SI Section).

The mixed-effect models of the three hormones and the skin temperature-based RQA metrics are shown in Table 3. No significant (*p* > 0.05) associations were observed between the leptin or cortisol and any of the RQA metrics as calculated from either the skin or the personal air sensor datasets. However, there were marginal associations in the skin temperature dataset between adiponectin and LAM (beta = 0.24, 95%CI: −0.02, 0.50, *p* = 0.065) and with DET (beta = 0.23, 95%CI: −0.035, 0.50, *p* = 0.087) (Table 3). For the personal air dataset, there were marginal associations between adiponectin and Lmean (beta = −0.31, 95%CI: −0.597, −0.031, *p* = 0.030) and with DIV (beta = −0.22, 95%CI: −0.456, −0.016, *p* = 0.067) (SI Section).

## 4. Discussion

This was an additional analysis of the original TEMP trial [10,11] that used wearable sensors to measure diurnal (24-h long, one-minute intervals) skin temperature temporal profiles of healthy, non-obese individuals crossing from one setting to another (urban to mountainous and vice versa) after a 7-day long stay in each setting during the Mediterranean summer. A notable difference of about 8–10 °C in daily max air temperature between the urban and mountainous settings during the study period was observed using governmental station measurement data. Skin temperature sensors generated data between 12,240 and 27,000 time point counts. We found dysregulations in skin temperature temporal dynamics of the study population while residing in the urban setting when compared with the cooler mountainous setting in Cyprus. These dysregulations took the form of reduced cycle duration and complexity, while skin temperature dynamics became less responsive to perturbations and less regular in magnitude. Simply by looking at the respective median skin temperatures in the two settings, it was obvious they were quite similar (35.1 °C [34.6, 35.6] vs. 35.05 °C [34.55, 35.7]) [10]. However, the RQA analysis offered an alternative perspective in inferring about the temporal dynamics of the skin temperature datasets by showing that the short-term (7-day) stay in an urban setting during the hot Mediterranean summer tended to blunt the cyclical properties of skin temperature diurnal dynamics observed for the same individuals in the cooler climate of the mountainous setting.

Skin temperature is an interesting “interface-variable” between the core body and ambient temperature regimes [34], being a faster responder to changes in ambient air temperature than that of core body temperature. As such, it may be the preferred marker of truly experienced temperature gradients at the individual level, in the absence of core body temperature measurements. There were indeed differences in the ambient air temperature levels between the two selected urban and mountainous settings in the summer, where the median [interquartile range, IQR] personal air temperature in the mountainous settings was 1.5 °C lower than that in the urban settings (27.1 °C [25.4, 29.2] vs. 28.6 °C [27.1, 30.5], *p* < 0.001), being consistent with the Mediterranean climate [10]. The cooler ambient air temperature profile of the mountainous setting vs. that of the urban setting during the summer period was confirmed with data obtained from the Cyprus Meteorological Service; data showed that Nicosia city (35 °C) > Limassol city (33 °C) > Troodos mountainous area (25 °C). Skin temperature seems to integrate the observed ambient air temperature gradient together with the time activity patterns of the study participants during the monitoring period in the respective setting.

Although heart rate and skin temperature temporal dynamics in a 24 h period are inversely related, there are similarities in the observed skin temperature sensitivity to human mobility and changes in environmental setting with those perturbations documented for heartbeat regulation in response to environmental stimuli as age progresses, where heart rate dynamics become increasingly predictable (constrained) on a beat-to-beat basis [21]. Moving beyond the traditional analyses of temperature time series with daily max, daily mean and regression models, this nonlinear RQA approach offers a new perspective on the characterization of the temperature regime experienced by individuals in different settings during a 24 h period. Skin temperature temporal dynamics in the urban setting were also characterized by lower entropy, i.e., lower temperature network complexity when compared with the more complex network cycles of skin temperature in the mountainous setting. Cycle regularity and response to perturbation as expressed by DET were also evidently reduced in the urban setting than those observed in the mountainous setting. This may be characteristic of the time activity patterns of individuals in the urban setting, where changes in different microenvironments of variable ambient temperatures and different activities during the day than those experienced in the more relaxing environment of the mountainous setting may be observed. Indeed, a higher number of temperature peaks was found in indoor (micro)environments than outdoors, suggesting higher fluctuations in personal air temperature levels across different indoor (micro)environments [10]. In this respect, it is worth noting that a more efficiently regulated system in physiology corresponds to a more sensitive system (and thus, more rapid adaptation) to environmental changes; this is, for example, the case with heartbeat dynamics [21] showing a marked increase in stability (higher recurrence) and decrease in complexity with increasing age. This situation mirrors what was observed in this study, with a marked decrease in complexity in urban settings with respect to mountainous settings.

Circadian and metabolic systems operate in synchronization in healthy individuals, where entraining agents of circadian rhythm, such as light, feeding, temperature and circadian markers (e.g., melatonin, cortisol, etc.) [35] are vital components of synchronized circadian systems [36]. Capturing the temporally dynamic process of dysregulation in biological systems is the key to better understanding the changes and processes undermining the state(s) of synchronization and resilience in biological systems. For example, the monitoring of perinatal temporal dynamics in the metabolism of heavy metals in children was able to predict well the emergence of autism spectrum disorder cases using the RQA algorithm and its metrics [28].

Observational studies often utilize centrally sited air temperature data from meteorological stations, where exposure misclassification bias is a concern. To accurately depict reliable individual-based dynamics of exposure/effect associations, a sufficiently large number of time-repeated samples per subject in the disease-relevant etiologic time interval, confined in a relatively long time series relative to the variation of the outcomes of interest, is warranted. Missing the right scale to look for biological rhythms is by far the most common methodological error in chrono-observations. This error stems from both epistemological faults and practical limitations, the two most relevant sources of error being: (i) the idea that the external *zeitgeiber* (e.g., day/night cycle) is the only source of biological rhythms without any consideration of endogenous sources. This consideration makes largely irrelevant the detection of rhythmic activity at the individual level and pushes towards aggregate-level population studies in which the variable of interest (e.g., a biomarker) is measured at few external conditions (e.g., morning/evening) considering only two time-points per subject; and (ii) the inherent difficulty of obtaining a sufficiently rich time series at the individual level. The most common bias is the time-fluctuating amplitude of the cyclic process, where in most cases, the most relevant point to address is the modification of the temporal correlation structure (e.g., increase/decrease in determinism or harmonic character of the time series). While this consciousness is widespread in physiology (e.g., heartbeat time series) [21], this is only in its infancy for environmental epidemiology studies. Recurrence analysis may detect state changes in complex biological systems, with this being practiced for more than three decades in a vast array of scientific disciplines (physiology, mathematics, physics, biology, medicine, neurology, pathology), but never applied before in the environmental health sciences [29,30,31].

A strength of this study was the multidisciplinary approach taken in coupling knowledge and information obtained from the RQA novel application in environmental health sciences and further coupling them with classical metabolic and stress hormone measurements. The randomized design is another strength of the study, together with the use of a wearable sensor, which is also novel method in behavioural change trials. Sensor data collection was continuous at the individual level, overcoming the exposure measurement error associated with temperature measurements obtained from a few specific discrete time points or measuring stations. Other strengths include the repeated measures and the multivariable adjustment. This study has few limitations. The sample size was small due to its proof-of-concept mode. A wide time window of several hours during the collection of the first morning void sample introduced additional variability in the magnitude of metabolic hormone diurnal patterns that was not accounted for.

## 5. Conclusions

The urban overheating phenomenon in Mediterranean cities is a societal challenge with vast implications for the protection of public health. The study documented the applicability of the RQA algorithms in delineating the temporal dynamic patterns of skin temperature as a surrogate of body core temperature, though in closer communication with alterations in external environmental stimuli that could affect skin temperature. The monitoring of personal air temperature fluctuations for urban dwellers may be key in preventing health-associated illnesses, by focusing on early-stage disease process that open up avenues for breakthroughs in precision health applications. The influence of desynchronization on an individual’s temporal health dynamics will help to adapt everyday lifestyles for effective disease prevention and wellbeing in the future. Accounting for individual circadian rhythms and personalized knowledge on temperature profiling during the warmer months are the next steps forward for precision exposome and its tools (e.g., sensors) that could be used to offer individualized heat protection and control advice. Differential sensitivities to heat stress by different age groups and differential critical life stage windows may pose a health risk to sensitive individuals; thus, stratified risk assessment approaches can take these susceptible groups into consideration and improve public health.

## Figures and Tables

**Table 1:** Demographics and baseline characteristics of the study population (overall, and by group).

	Overall	First Urban	First Mountainous	*p* Value *
n	14	8	6	
Age (mean (SD))	42.00 (9.11)	40.86 (8.01)	43.33 (10.88)	NA
Sex = Male (%)	3 (21.4)	2 (25.0)	1 (16.7)	1.000
BMI (mean (SD))	24.94 (3.22)	25.89 (3.67)	23.68 (2.19)	NA
BMI category = overweight (%)	5 (35.7)	4 (50.0)	1 (16.7)	0.469
Educational level (%)				0.161
Secondary	5 (35.7)	3 (37.5)	2 (33.3)	
University/college	3 (21.4)	3 (37.5)	0 (0.0)	
Master/PhD	6 (42.9)	2 (25.0)	4 (66.7)	
Chronotype ^ (%)				0.117
early	4 (40.0)	4 (57.1)	0 (0.0)	
intermediate	5 (50.0)	2 (28.6)	3 (100.0)	
late	1 (10.0)	1 (14.3)	0 (0.0)	
Smoking status (%)				0.239
Smoker	2 (14.3)	2 (25.0)	0 (0.0)	
Non-smoker	11 (78.6)	5 (62.5)	6 (100.0)	
Former smoker	1 (7.1)	1 (12.5)	0 (0.0)	
Alcohol frequency (%)				0.823
Weekly	3 (21.4)	2 (25.0)	1 (16.7)	
Monthly	3 (21.4)	2 (25.0)	1 (16.7)	
Rarely/never	8 (57.1)	4 (50.0)	4 (66.7)	
Physical exercise = No (%)	7 (50.0)	7 (87.5)	0 (0.0)	0.007
Screen time (hr/day) (mean (SD))	5.36 (3.25)	5.88 (3.48)	4.67 (3.08)	NA
Days in mountainous setting (mean (SD))	7.71 (3.10)	7.00 (1.20)	8.67 (4.59)	NA
Washout days (mean (SD))	11.88 (7.28)	11.88 (7.28)	NA	NA

* the above variables were tested for differences between the two groups by chi-square tests for categorical variables and *t*-tests for normally distributed continuous variables. ^ the chronotype category cut-offs are: early (<3:00), intermediate (3:00–5:00) and late (>5:00). [29].

**Table 2:** Linear mixed-effect models of log-transformed RQA metrics as a function of mountainous setting (in comparison to the urban setting) accounting for the repeated measurements.

RQA	Term	Estimate	Std.Error	*p* Value	Lower Bound	Upper Bound
Lmean	Mountainous	0.841	0.232	0.0006	0.384	1.297
Vmean	Mountainous	0.83	0.231	0.00069	0.380	1.286
DET	Mountainous	0.780	0.230	0.00139	0.325	1.228
PC1	Mountainous	0.745	0.224	0.00160	0.306	1.185
DIV	Mountainous	−0.652	0.211	0.00319	−1.106	−0.238
LAM	Mountainous	0.764	0.231	0.00170	0.311	1.217
ENT	Mountainous	0.707	0.243	0.00500	0.232	1.182
TRE	Mountainous	−0.322	0.236	0.17777	−0.785	0.140
REC	Mountainous	0.181	0.253	0.477567	−0.314	0.676

**Table 3:** Linear mixed-effect models of log-transformed biomarkers (leptin, adiponectin and cortisol) as a function of first morning sample (in comparison to the last-before-sleep sample) accounting for the repeated measurements and for age.

Leptin					
RQA Metric	Estimate	Std.Error	*p*-Value	Lower Bound	Upper Bound
TREND	0.214	0.141	0.1358313	−0.069	0.497
Lmean	−0.129	0.128	0.3171442	−0.387	0.128
LAM	0.121	0.128	0.3483536	−0.136	0.380
Vmean	−0.114	0.128	0.3812657	−0.372	0.144
DET	0.102	0.131	0.4397261	−0.161	0.365
DIV	−0.083	0.142	0.5616446	−0.368	0.202
PC1	0.073	0.131	0.5808591	−0.191	0.337
REC	0.039	0.129	0.7627006	−0.220	0.299
ENT	−0.013	0.129	0.9180662	−0.273	0.246
**Adiponectin**					
**RQA Metric**	**Estimate**	**Std.Error**	***p*** **-Value**	**Lower Bound**	**Upper Bound**
LAM	0.246	0.130	0.0647188	-0.015	0.507
DET	0.233	0.133	0.0871753	−0.035	0.501
REC	0.191	0.133	0.1562231	−0.075	0.459
PC1	0.175	0.136	0.2050513	−0.098	0.449
ENT	0.122	0.134	0.3678370	−0.147	0.391
DIV	−0.132	0.148	0.3747916	−0.430	0.164
TREND	0.111	0.148	0.4552794	−0.186	0.409
Lmean	−0.030	0.135	0.8276175	−0.302	0.242
Vmean	0.006	0.136	0.9606054	−0.266	0.279
**Cortisol**					
**RQA Metric**	**Estimate**	**Std.Error**	***p*** **-Value**	**Lower Bound**	**Upper Bound**
Vmean	0.101	0.128	0.4343851	−0.157	0.361
ENT	0.091	0.127	0.4763856	−0.165	0.348
Lmean	0.080	0.128	0.5329737	−0.178	0.339
LAM	0.068	0.129	0.5995378	−0.192	0.329
DET	0.066	0.130	0.6145308	−0.196	0.328
PC1	0.067	0.134	0.6177130	−0.201	0.336
TREND	−0.028	0.137	0.8357969	−0.307	0.249
DIV	0.025	0.144	0.8621418	−0.264	0.315
REC	0.017	0.131	0.8923230	−0.246	0.281

Model details: Leptin, adiponectin and cortisol are creatinine adjusted and log transformed. Random intercepts for the repeated samples within participants with unstructured covariance matrix.

## Data Availability

The authors commit to making the relevant anonymized individual-level data available via open access here: https://doi.org/10.5281/zenodo.7329878.

## Supplementary Materials

The following supporting information can be downloaded at: https://www.mdpi.com/article/10.3390/ijerph192215195/s1.

## References

[1] Myers S.S. (2017). Planetary health: Protecting human health on a rapidly changing planet. Lancet.

[2] Sanchez M.G., de’ Donato F.K.V. (2021). Heat and Health in the WHO European Region: Updated Evidence for Effective Prevention.

[3] World Health Organization (2018). Report of the systematic review on the effect of indoor heat on health. WHO Housing and Health Guidelines.

[4] World Health Organization (2017). Climate Change and Health—Fact Sheet. www.who.int/mediacentre/factsheets/fs266/en.

[5] Uejio C.K., Tamerius J.D., Vredenburg J., Asaeda G., Isaacs D.A., Braun J., Quinn A., Freese J.P. (2016). Summer indoor heat exposure and respiratory and cardiovascular distress calls in New York City, NY, U.S. Indoor Air.

[6] Wallace L. (1996). Indoor Particles: A Review. J. Air Waste Manag. Assoc..

[7] Armstrong B.K., Saracci R., White E. (1992). Principles of Exposure Measurement in Epidemiology.

[8] Zeger S.L., Thomas D., Dominici F., Samet J.M., Schwartz J., Dockery D., Cohen A. (2000). Exposure Measurement Error in Time-Series Studies of Air Pollution: Concepts and Consequences. Env. Health Persp..

[9] Thomas D., Stram D., Dwyer J. (1993). Exposure measurement error: Influence on exposure-disease relationships and methods of correction. Annu. Rev. Public Health.

[10] Constantinou A., Oikonomou S., Konstantinou C., Makris K.C. (2021). A randomized cross-over trial investigating differences in 24-h personal air and skin temperatures using wearable sensors between two climatologically contrasting settings. Sci. Rep..

[11] Makris K.C., Konstantinou C., Perikkou A., Zdravic A.B., Christophi C.A. (2020). Contrasting short-term temperature effects on the profiling of metabolic and stress hormones in non-obese healthy adults: A randomized cross-over trial. Environ. Res..

[12] Haddad N., Andrianou X.D., Makris K.C. (2019). A Scoping Review on the Characteristics of Human Exposome Studies. Curr. Pollut. Rep..

[13] Klepeis N.E., Nelson W.C., Ott W.R., Robinson J.P., Tsang A.M., Switzer P., Behar J.V., Hern S.C., Engelmann W.H. (2001). The National Human Activity Pattern Survey (NHAPS): A resource for assessing exposure to environmental pollutants. J. Expo. Sci. Environ. Epidemiol..

[14] Makris K.C. (2021). Desynchronized circadian clock and exposures to xenobiotics are associated with differentiated disease phenotypes. BioEssays.

[15] Zheng Z. (2022). Collective Sustained Oscillations in Complex Systems. Recent Trends in Chaotic, Nonlinear and Complex Dynamics.

[16] Tiwari S.P., Reuter N. (2018). Conservation of intrinsic dynamics in proteins—What have computational models taught us?. Curr. Opin. Struct. Biol..

[17] Tzuk O., Ujjwal S.R., Fernandez-Oto C., Seifan M., Meron E. (2019). Interplay between exogenous and endogenous factors in seasonal vegetation oscillations. Sci. Rep..

[18] Arora M., Curtin P., Curtin A., Austin C., Giuliani A. (2022). Environmental Biodynamics: A New Science of How the Environment Interacts with Human Health.

[19] Shamsan A., Wu X., Liu P., Cheng C. (2020). Intrinsic recurrence quantification analysis of nonlinear and nonstationary short-term time series. Chaos: Interdiscip. J. Nonlinear Sci..

[20] Smith T.J., Kriebel D. (2010). A Biologic Approach to Environmental Assessment and Epidemiology.

[21] Giuliani A., Piccirillo G., Marigliano V., Colosimo A. (1998). A nonlinear explanation of aging-induced changes in heartbeat dynamics. Am. J. Physiol.-Heart Circ. Physiol..

[22] Rampichini S., Vieira T.M., Castiglioni P., Merati G. (2020). Complexity Analysis of Surface Electromyography for Assessing the Myoelectric Manifestation of Muscle Fatigue: A Review. Entropy.

[23] Ouyang G., Li X., Dang C., Richards D.A. (2008). Using recurrence plot for determinism analysis of EEG recordings in genetic absence epilepsy rats. Clin. Neurophysiol..

[24] Giuliani A., Benigni R., Zbilut J.P., Webber C.L., Sirabella P., Colosimo A. (2002). Nonlinear Signal Analysis Methods in the Elucidation of Protein Sequence−Structure Relationships. Chem. Rev..

[25] Trulla L.L., Giuliani A., Zbilut J.P., Webber C.L. (1996). Recurrence quantification analysis of the logistic equation with transients. Phys. Lett. A.

[26] Wallot S., Leonardi G. (2018). Analyzing Multivariate Dynamics Using Cross-Recurrence Quantification Analysis (CRQA), Diagonal-Cross-Recurrence Profiles (DCRP), and Multidimensional Recurrence Quantification Analysis (MdRQA)—A Tutorial in R. Front. Psychol..

[27] Facchini A., Mocenni C., Marwan N., Vicino A., Tiezzi E. (2007). Nonlinear time series analysis of dissolved oxygen in the Orbetello Lagoon (Italy). Ecol. Model..

[28] Curtin P., Austin C., Curtin A., Gennings C., Arora M., Tammimies K., Willfors C., Berggren S., Siper P., Rai D. (2018). Dynamical features in fetal and postnatal zinc-copper metabolic cycles predict the emergence of autism spectrum disorder. Sci. Adv..

[29] Marwan N., Riley M., Giuliani A., Webber C.L. (2013). Translational Recurrences: From Mathematical Theory to Real-World Applications. Proceedings of the 5th International Symposium on Recurrence Plots, Chicago, IL, USA, 14–16 August 2013.

[30] Marwan N., Webber C.L., Webber C., Marwan N. (2015). Mathematical and Computational Foundations of Recurrence Quantifications. Recurrence Quantification Analysis: Theory and Best Practices.

[31] Webber C.L., Marwan N., Facchini A., Giuliani A. (2009). Simpler methods do it better: Success of Recurrence Quantification Analysis as a general-purpose data analysis tool. Phys. Lett. A.

[32] Moon J.-Y., Jung H.-J., Moon M.H., Chung B.C., Choi M.H. (2009). Heat-map visualization of gas chromatography-mass spectrometry based quantitative signatures on steroid metabolism. J. Am. Soc. Mass Spectrom..

[33] (2010). The MAK-Collection for Occupational Health and Safety, Part IV: Biomonitoring Methods.

[34] Martinez-Nicolas A., Meyer M., Hunkler S., Madrid J.A., Rol M.A., Meyer A.H., Schötzau A., Orgül S., Kräuchi K. (2015). Daytime variation in ambient temperature affects skin temperatures and blood pressure: Ambulatory winter/summer comparison in healthy young women. Physiol. Behav..

[35] Dallmann R., Okyar A., Lévi F. (2016). Dosing-Time Makes the Poison: Circadian Regulation and Pharmacotherapy. Trends Mol. Med..

[36] Phillips M.L. (2009). Circadian rhythms: Of owls, larks and alarm clocks. Nature.

