# Supplementary material for: Diurnal Nonlinear Recurrence Metrics of Skin Temperature and Their Association with Metabolic Hormones in Contrasting Climate Settings: A Randomized Cross-Over Trial
Source: Int J Environ Res Public Health. 2022 Nov 17;19(22):15195. doi: 10.3390/ijerph192215195 (PMC9690349; doi:10.3390/ijerph192215195)
Supplement: Supplementary file 1 [file ijerph-19-15195-s001.zip › SI_section_RQA analysis_MakrisKC (1).html]

RQA analysis skin temperature


## RQA analysis skin temperature

# RQA analysis skin temperature

#### Nikolaos Efthymiou

#### 16/11/2022

**Participants:**- We have 106 temperature measurements
- 53 for skin and 53 for air temperature
- In those 53 skin temperature files we have a total of 37 participants
- We drop 21 participants because they dont have temperature measurements
  in both settings (16 participants left)
- We drop 1 participant because of invalid biomarker measurements (15
  participants left)
- We drop 1 participant because of faulty skin temperature measurements
  (14 participants left)

|  | First urban (N=8) | First mountainous (N=6) | Overall (N=14) | P-value |
| --- | --- | --- | --- | --- |
| Age |  |  |  |  |
| Mean (SD) | 42.0 (7.80) | 45.3 (10.9) | 43.4 (9.01) | 0.54 |
| Median [Min, Max] | 39.5 [32.0, 56.0] | 44.5 [31.0, 60.0] | 40.5 [31.0, 60.0] |  |
| Sex |  |  |  |  |
| Female | 6 (75.0%) | 5 (83.3%) | 11 (78.6%) | 1 |
| Male | 2 (25.0%) | 1 (16.7%) | 3 (21.4%) |  |
| BMI |  |  |  |  |
| Mean (SD) | 25.9 (3.67) | 23.7 (2.19) | 24.9 (3.22) | 0.186 |
| Median [Min, Max] | 26.0 [19.4, 29.8] | 24.0 [21.0, 27.2] | 24.6 [19.4, 29.8] |  |
| BMIcat |  |  |  |  |
| underweight | 0 (0%) | 0 (0%) | 0 (0%) | 0.301 |
| normal weight | 4 (50.0%) | 5 (83.3%) | 9 (64.3%) |  |
| overweight | 4 (50.0%) | 1 (16.7%) | 5 (35.7%) |  |
| education\_level |  |  |  |  |
| Secondary | 3 (37.5%) | 2 (33.3%) | 5 (35.7%) | 0.199 |
| University/college | 3 (37.5%) | 0 (0%) | 3 (21.4%) |  |
| Master/PhD | 2 (25.0%) | 4 (66.7%) | 6 (42.9%) |  |
| Smoking\_status |  |  |  |  |
| Smoker | 2 (25.0%) | 0 (0%) | 2 (14.3%) | 0.473 |
| Non-smoker | 5 (62.5%) | 6 (100%) | 11 (78.6%) |  |
| Former smoker | 1 (12.5%) | 0 (0%) | 1 (7.1%) |  |
| alcohol\_freq |  |  |  |  |
| Weekly | 2 (25.0%) | 1 (16.7%) | 3 (21.4%) | 1 |
| Monthly | 2 (25.0%) | 1 (16.7%) | 3 (21.4%) |  |
| Rarely/Never | 4 (50.0%) | 4 (66.7%) | 8 (57.1%) |  |
| Physical\_exercise |  |  |  |  |
| Yes | 1 (12.5%) | 6 (100%) | 7 (50.0%) | 0.00466 |
| No | 7 (87.5%) | 0 (0%) | 7 (50.0%) |  |
| screen\_hours\_day |  |  |  |  |
| Mean (SD) | 5.88 (3.48) | 4.67 (3.08) | 5.36 (3.25) | 0.506 |
| Median [Min, Max] | 6.50 [2.00, 10.0] | 4.00 [1.00, 10.0] | 5.00 [1.00, 10.0] |  |
| days\_mountain |  |  |  |  |
| Mean (SD) | 7.00 (1.20) | 8.67 (4.59) | 7.71 (3.10) | 0.422 |
| Median [Min, Max] | 7.50 [5.00, 8.00] | 6.50 [5.00, 15.0] | 7.00 [5.00, 15.0] |  |
| washout\_days |  |  |  |  |
| Mean (SD) | 11.9 (7.28) | 19.7 (8.41) | 15.2 (8.47) | 0.0995 |
| Median [Min, Max] | 14.0 [2.00, 22.0] | 22.5 [6.00, 30.0] | 15.0 [2.00, 30.0] |  |

RQA analysis Settings: time.lag=1, radius = 0.1


| ID | setting | REC | DET | DIV | Lmean | ENTR | TREND | LAM | Vmean |
| --- | --- | --- | --- | --- | --- | --- | --- | --- | --- |
| T10-U | Urban | 0.0595910 | 0.9034054 | 0.0062893 | 5.272873 | 2.170489 | -0.0000926 | 0.9309530 | 5.914143 |
| T11-U | Urban | 0.0650993 | 0.8843914 | 0.0064516 | 4.776698 | 2.092872 | 0.0000086 | 0.9153641 | 5.793558 |
| T12-U | Urban | 0.0621634 | 0.9180152 | 0.0038023 | 5.571018 | 2.297317 | -0.0000843 | 0.9411724 | 6.731343 |
| T16-U | Urban | 0.0432359 | 0.9003279 | 0.0031250 | 6.267412 | 2.273365 | -0.0000715 | 0.9145381 | 7.318101 |
| T17-U | Urban | 0.0757774 | 0.9237711 | 0.0050761 | 5.521893 | 2.334194 | -0.0000462 | 0.9478400 | 6.915997 |
| T24-U | Urban | 0.0794569 | 0.8956582 | 0.0129870 | 4.397663 | 2.016300 | -0.0000939 | 0.9252764 | 4.898158 |
| T26-U | Urban | 0.0244792 | 0.8188731 | 0.0116279 | 4.133864 | 1.819283 | -0.0000072 | 0.8498030 | 4.404779 |
| T29-U | Urban | 0.0451341 | 0.9230260 | 0.0032895 | 5.883403 | 2.378324 | -0.0000155 | 0.9484774 | 6.916089 |
| T31-U | Urban | 0.0396316 | 0.8543441 | 0.0081967 | 4.765492 | 2.019598 | -0.0000284 | 0.8879654 | 5.297111 |
| T33-U | Urban | 0.0455633 | 0.8959992 | 0.0068966 | 5.050653 | 2.145141 | -0.0000423 | 0.9290008 | 5.862801 |
| T34-U | Urban | 0.0361323 | 0.8750200 | 0.0129870 | 4.735284 | 2.041002 | 0.0000066 | 0.9011265 | 5.056999 |
| T35-U | Urban | 0.0987288 | 0.9625935 | 0.0030960 | 7.659295 | 2.741629 | -0.0001313 | 0.9741750 | 10.000351 |
| T45-U | Urban | 0.0472820 | 0.8877647 | 0.0086957 | 4.703594 | 2.090574 | -0.0000031 | 0.9163335 | 5.203348 |
| T60-U | Urban | 0.0442332 | 0.9341270 | 0.0048077 | 6.257212 | 2.461850 | -0.0000105 | 0.9520944 | 7.635569 |
| T10-M | Mountainous | 0.0938204 | 0.9878589 | 0.0008905 | 20.725116 | 3.670949 | -0.0001596 | 0.9841323 | 26.826258 |
| T11-M | Mountainous | 0.0542544 | 0.8859931 | 0.0046083 | 5.185786 | 2.070951 | -0.0000825 | 0.9073261 | 5.521800 |
| T12-M | Mountainous | 0.0744223 | 0.9390884 | 0.0031949 | 8.004529 | 2.413887 | -0.0001078 | 0.9555021 | 9.686966 |
| T16-M | Mountainous | 0.0757996 | 0.9430709 | 0.0033784 | 6.666517 | 2.470191 | -0.0000866 | 0.9599181 | 8.390968 |
| T17-M | Mountainous | 0.0673148 | 0.9158500 | 0.0052356 | 5.059084 | 2.206857 | -0.0000597 | 0.9471644 | 6.024012 |
| T24-M | Mountainous | 0.0403665 | 0.8680350 | 0.0071942 | 4.623481 | 1.970126 | -0.0000177 | 0.9067548 | 5.149186 |
| T26-M | Mountainous | 0.0506211 | 0.9373523 | 0.0034843 | 6.333569 | 2.434794 | -0.0000446 | 0.9559485 | 8.096829 |
| T29-M | Mountainous | 0.0396402 | 0.9065914 | 0.0079365 | 5.523682 | 2.293040 | -0.0000481 | 0.9383318 | 6.547453 |
| T31-M | Mountainous | 0.0323573 | 0.8709610 | 0.0086207 | 4.903751 | 1.931763 | -0.0000481 | 0.9034965 | 5.584615 |
| T33-M | Mountainous | 0.0521518 | 0.9362320 | 0.0048077 | 7.061868 | 2.512756 | -0.0000821 | 0.9566403 | 8.586736 |
| T34-M | Mountainous | 0.0795986 | 0.9443622 | 0.0068493 | 6.284466 | 2.458174 | -0.0001414 | 0.9682935 | 7.576508 |
| T35-M | Mountainous | 0.0533700 | 0.9608920 | 0.0016863 | 17.447088 | 2.675620 | -0.0001034 | 0.9635306 | 16.787154 |
| T45-M | Mountainous | 0.0498987 | 0.9462646 | 0.0051282 | 9.016484 | 2.752232 | 0.0000103 | 0.9445926 | 9.687481 |
| T60-M | Mountainous | 0.0590673 | 0.9514051 | 0.0043103 | 7.610868 | 2.596223 | 0.0001027 | 0.9652520 | 9.489205 |

## RQA Plots

## PCA analysis

Eigenvalues/variances

|  | eigenvalue | variance.percent | cumulative.variance.percent |
| --- | --- | --- | --- |
| Dim.1 | 5.4822673 | 68.5283410 | 68.52834 |
| Dim.2 | 0.9926115 | 12.4076438 | 80.93598 |
| Dim.3 | 0.7990761 | 9.9884510 | 90.92444 |
| Dim.4 | 0.4334924 | 5.4186551 | 96.34309 |
| Dim.5 | 0.2028643 | 2.5358036 | 98.87889 |
| Dim.6 | 0.0693162 | 0.8664519 | 99.74535 |
| Dim.7 | 0.0171898 | 0.2148719 | 99.96022 |
| Dim.8 | 0.0031825 | 0.0397816 | 100.00000 |

Coordinates of variables

|  | Dim.1 | Dim.2 | Dim.3 | Dim.4 | Dim.5 |
| --- | --- | --- | --- | --- | --- |
| REC | 0.7191258 | -0.5713829 | 0.0761212 | 0.2664070 | -0.2802574 |
| DET | 0.9350897 | -0.0166398 | 0.3034802 | 0.0535048 | 0.1522846 |
| DIV | -0.7857237 | -0.1339225 | -0.2548890 | 0.5130884 | 0.1909462 |
| Lmean | 0.8423419 | 0.3599853 | -0.3689115 | 0.0533150 | -0.0018347 |
| ENTR | 0.9463790 | 0.2080984 | -0.0110779 | 0.1301327 | 0.0044391 |
| TREND | -0.5505767 | 0.6051605 | 0.4934000 | 0.2538726 | -0.1473390 |
| LAM | 0.8838002 | -0.1294330 | 0.3877210 | 0.0549202 | 0.1991669 |
| Vmean | 0.8854154 | 0.3034047 | -0.3259195 | 0.0956696 | -0.0571813 |

Contribution of variables

|  | Dim.1 | Dim.2 | Dim.3 | Dim.4 | Dim.5 |
| --- | --- | --- | --- | --- | --- |
| REC | 9.432992 | 32.8908551 | 0.7251415 | 16.3723064 | 38.7176161 |
| DET | 15.949472 | 0.0278944 | 11.5258375 | 0.6603948 | 11.4315825 |
| DIV | 11.261067 | 1.8068737 | 8.1304420 | 60.7299539 | 17.9728352 |
| Lmean | 12.942454 | 13.0554045 | 17.0316309 | 0.6557184 | 0.0016593 |
| ENTR | 16.336911 | 4.3627285 | 0.0153577 | 3.9065332 | 0.0097136 |
| TREND | 5.529367 | 36.8945212 | 30.4656300 | 14.8679160 | 10.7011399 |
| LAM | 14.247807 | 1.6877593 | 18.8126718 | 0.6957977 | 19.5536856 |
| Vmean | 14.299930 | 9.2739634 | 13.2932887 | 2.1113794 | 1.6117678 |

## RQA descriptives Overall and by setting

| metric | setting | mean | sd | q0 | q25 | q50 | q75 | q90 | q95 | q100 |
| --- | --- | --- | --- | --- | --- | --- | --- | --- | --- | --- |
| DET | Mountainous | 0.9281398 | 0.0345731 | 0.8680350 | 0.9089060 | 0.9382204 | 0.9457890 | 0.9580460 | 0.9703304 | 0.9878589 |
| DET | Overall | 0.9132598 | 0.0374829 | 0.8188731 | 0.8873218 | 0.9169326 | 0.9400840 | 0.9542512 | 0.9619980 | 0.9878589 |
| DET | Urban | 0.8983798 | 0.0352961 | 0.8188731 | 0.8852347 | 0.8981635 | 0.9217733 | 0.9310203 | 0.9440903 | 0.9625935 |
| Dim.1 | Mountainous | 0.9280890 | 2.5398309 | -2.4496847 | -0.6500129 | 0.9191682 | 1.5481476 | 3.1198800 | 5.0772192 | 7.6116908 |
| Dim.1 | Overall | 0.0000000 | 2.3843897 | -4.6524333 | -1.4027553 | 0.0300195 | 0.9897313 | 2.1528298 | 3.5061379 | 7.6116908 |
| Dim.1 | Urban | -0.9280890 | 1.8713647 | -4.6524333 | -1.7947273 | -0.8383016 | 0.1961672 | 0.4089401 | 1.3837977 | 3.1228869 |
| Dim.2 | Mountainous | 0.1535347 | 1.0895552 | -1.9085288 | -0.7424925 | 0.2666008 | 0.9198058 | 1.4817571 | 1.6400065 | 1.9204143 |
| Dim.2 | Overall | 0.0000000 | 1.0145811 | -2.0690360 | -0.7626121 | 0.2666008 | 0.6227819 | 1.2094341 | 1.4805470 | 1.9204143 |
| Dim.2 | Urban | -0.1535347 | 0.9486989 | -2.0690360 | -0.7296142 | 0.1626016 | 0.5424521 | 0.7964603 | 0.8544883 | 0.8884815 |
| DIV | Mountainous | 0.0048089 | 0.0022574 | 0.0008905 | 0.0034049 | 0.0047080 | 0.0064459 | 0.0077138 | 0.0081760 | 0.0086207 |
| DIV | Overall | 0.0058805 | 0.0030993 | 0.0008905 | 0.0034578 | 0.0051022 | 0.0073798 | 0.0095753 | 0.0125113 | 0.0129870 |
| DIV | Urban | 0.0069520 | 0.0035187 | 0.0030960 | 0.0040536 | 0.0063705 | 0.0085709 | 0.0125793 | 0.0129870 | 0.0129870 |
| ENTR | Mountainous | 2.4612545 | 0.4283064 | 1.9317634 | 2.2284027 | 2.4464839 | 2.5753558 | 2.7292485 | 3.0737832 | 3.6709492 |
| ENTR | Overall | 2.3335536 | 0.3616510 | 1.8192829 | 2.0856683 | 2.2951786 | 2.4639350 | 2.6954225 | 2.7485211 | 3.6709492 |
| ENTR | Urban | 2.2058527 | 0.2303769 | 1.8192829 | 2.0533950 | 2.1578154 | 2.3249750 | 2.4367919 | 2.5597724 | 2.7416289 |
| LAM | Mountainous | 0.9469203 | 0.0248347 | 0.9034965 | 0.9398970 | 0.9557253 | 0.9626274 | 0.9673811 | 0.9738371 | 0.9841323 |
| LAM | Overall | 0.9353930 | 0.0298962 | 0.8498030 | 0.9151576 | 0.9428825 | 0.9561214 | 0.9661645 | 0.9721165 | 0.9841323 |
| LAM | Urban | 0.9238657 | 0.0308766 | 0.8498030 | 0.9147446 | 0.9271386 | 0.9461731 | 0.9510093 | 0.9598226 | 0.9741750 |
| Lmean | Mountainous | 8.1747350 | 4.8357655 | 4.6234808 | 5.2702604 | 6.5000431 | 7.9061139 | 14.9179067 | 18.5943976 | 20.7251159 |
| Lmean | Overall | 6.7658087 | 3.7062986 | 4.1338637 | 4.8719876 | 5.5473504 | 6.7653545 | 8.3081156 | 14.4963765 | 20.7251159 |
| Lmean | Urban | 5.3568823 | 0.9326268 | 4.1338637 | 4.7428357 | 5.1617633 | 5.8053065 | 6.2643520 | 6.7545711 | 7.6592950 |
| REC | Mountainous | 0.0587631 | 0.0173453 | 0.0323573 | 0.0500793 | 0.0538122 | 0.0726454 | 0.0784589 | 0.0845762 | 0.0938204 |
| REC | Overall | 0.0567568 | 0.0184224 | 0.0244792 | 0.0439839 | 0.0527609 | 0.0690917 | 0.0794994 | 0.0888428 | 0.0987288 |
| REC | Urban | 0.0547506 | 0.0198834 | 0.0244792 | 0.0434852 | 0.0464226 | 0.0643654 | 0.0783531 | 0.0862021 | 0.0987288 |
| TREND | Mountainous | -0.0000620 | 0.0000657 | -0.0001596 | -0.0000992 | -0.0000709 | -0.0000455 | 0.0000019 | 0.0000427 | 0.0001027 |
| TREND | Overall | -0.0000529 | 0.0000557 | -0.0001596 | -0.0000881 | -0.0000481 | -0.0000142 | 0.0000072 | 0.0000097 | 0.0001027 |
| TREND | Urban | -0.0000437 | 0.0000442 | -0.0001313 | -0.0000811 | -0.0000353 | -0.0000080 | 0.0000037 | 0.0000073 | 0.0000086 |
| Vmean | Mountainous | 9.5682266 | 5.7641164 | 5.1491859 | 6.1548726 | 8.2438985 | 9.6375258 | 14.6572520 | 20.3008400 | 26.8262575 |
| Vmean | Overall | 7.9251257 | 4.4508833 | 4.4047789 | 5.5689116 | 6.8236702 | 8.4399103 | 9.7813423 | 14.4117727 | 26.8262575 |
| Vmean | Urban | 6.2820248 | 1.4507410 | 4.4047789 | 5.2267884 | 5.8884723 | 6.9160659 | 7.5403283 | 8.4632425 | 10.0003510 |

## Paired T test after log transformation for each variable for the 2 groups (alternative hypothesis: true difference in means is not equal to 0)

Paired - T test


| metric | estimate | statistic | p.value | parameter | conf.low | conf.high | method | alternative |
| --- | --- | --- | --- | --- | --- | --- | --- | --- |
| REC | -0.0038366 | -0.5848616 | 0.5686533 | 13 | -0.0180081 | 0.0103350 | Paired t-test | two.sided |
| DET | -0.0155664 | -2.6571506 | 0.0197413 | 13 | -0.0282224 | -0.0029103 | Paired t-test | two.sided |
| DIV | 0.0021272 | 2.3817886 | 0.0331968 | 13 | 0.0001978 | 0.0040567 | Paired t-test | two.sided |
| Lmean | -0.2849791 | -2.9412937 | 0.0114620 | 13 | -0.4942952 | -0.0756631 | Paired t-test | two.sided |
| ENTR | -0.0724921 | -2.2339090 | 0.0436852 | 13 | -0.1425977 | -0.0023864 | Paired t-test | two.sided |
| TREND | 0.0000184 | 1.0564493 | 0.3100008 | 13 | -0.0000192 | 0.0000559 | Paired t-test | two.sided |
| LAM | -0.0119568 | -2.4443546 | 0.0295245 | 13 | -0.0225245 | -0.0013891 | Paired t-test | two.sided |
| Vmean | -0.2955314 | -2.8728463 | 0.0130716 | 13 | -0.5177698 | -0.0732930 | Paired t-test | two.sided |
| Dim.1 | -0.3663775 | -2.4067249 | 0.0316836 | 13 | -0.6952521 | -0.0375030 | Paired t-test | two.sided |

## Leptin,cortisol and adiponectin are adjusted for creatinine and then log transformed (Metrics are log transformed in a previous step)

## Leptin models

##### Formula:leptin ~ Mesurment + SampleType + Age + (1 | ID) Number of observations:56

p.values values for Metabolites (14 total models)


| MesurmentName | effect | term | estimate | std.error | statistic | df | p.value | conf.low | conf.high |
| --- | --- | --- | --- | --- | --- | --- | --- | --- | --- |
| TREND | fixed | Mesurment | 0.2117733 | 0.1397831 | 1.5150138 | 51.93998 | 0.1358313 | -0.0687297 | 0.4922763 |
| Lmean | fixed | Mesurment | -0.1287979 | 0.1274881 | -1.0102738 | 50.88418 | 0.3171442 | -0.3847552 | 0.1271593 |
| LAM | fixed | Mesurment | 0.1207388 | 0.1275635 | 0.9464999 | 51.03760 | 0.3483536 | -0.1353509 | 0.3768285 |
| Vmean | fixed | Mesurment | -0.1128583 | 0.1277718 | -0.8832799 | 50.56105 | 0.3812657 | -0.3694250 | 0.1437084 |
| DET | fixed | Mesurment | 0.1011901 | 0.1299490 | 0.7786914 | 51.48210 | 0.4397261 | -0.1596340 | 0.3620142 |
| DIV | fixed | Mesurment | -0.0826193 | 0.1414056 | -0.5842719 | 50.38055 | 0.5616446 | -0.3665877 | 0.2013490 |
| Dim.1 | fixed | Mesurment | 0.0724496 | 0.1303585 | 0.5557716 | 49.72798 | 0.5808591 | -0.1894187 | 0.3343179 |
| REC | fixed | Mesurment | 0.0392600 | 0.1293219 | 0.3035836 | 50.17184 | 0.7627006 | -0.2204687 | 0.2989887 |
| ENTR | fixed | Mesurment | -0.0132578 | 0.1282431 | -0.1033804 | 51.04260 | 0.9180662 | -0.2707114 | 0.2441958 |

## Adiponectin models

##### Formula:adiponectin ~ Mesurment + SampleType + Age + (1 | ID) Number of observations:56

p.values values for Metabolites (14 total models)


| MesurmentName | effect | term | estimate | std.error | statistic | df | p.value | conf.low | conf.high |
| --- | --- | --- | --- | --- | --- | --- | --- | --- | --- |
| LAM | fixed | Mesurment | 0.2455602 | 0.1301070 | 1.8873720 | 51.80246 | 0.0647188 | -0.0155422 | 0.5066626 |
| DET | fixed | Mesurment | 0.2312141 | 0.1326227 | 1.7433976 | 51.99182 | 0.0871754 | -0.0349138 | 0.4973419 |
| REC | fixed | Mesurment | 0.1915353 | 0.1331008 | 1.4390247 | 51.25184 | 0.1562231 | -0.0756438 | 0.4587144 |
| Dim.1 | fixed | Mesurment | 0.1736237 | 0.1352579 | 1.2836490 | 51.13568 | 0.2050513 | -0.0979004 | 0.4451479 |
| ENTR | fixed | Mesurment | 0.1209754 | 0.1331673 | 0.9084468 | 51.95502 | 0.3678370 | -0.1462498 | 0.3882006 |
| DIV | fixed | Mesurment | -0.1321352 | 0.1475871 | -0.8953033 | 51.48750 | 0.3747916 | -0.4283607 | 0.1640902 |
| TREND | fixed | Mesurment | 0.1107087 | 0.1471355 | 0.7524264 | 50.62191 | 0.4552794 | -0.1847318 | 0.4061491 |
| Lmean | fixed | Mesurment | -0.0294941 | 0.1347621 | -0.2188602 | 51.88779 | 0.8276175 | -0.2999279 | 0.2409398 |
| Vmean | fixed | Mesurment | 0.0067009 | 0.1350064 | 0.0496336 | 51.77543 | 0.9606054 | -0.2642373 | 0.2776390 |

## Cortisol models

##### Formula:cortisol ~ Mesurment + SampleType + Age + (1 | ID) Number of observations:56

p.values values for Metabolites (14 total models)


| MesurmentName | effect | term | estimate | std.error | statistic | df | p.value | conf.low | conf.high |
| --- | --- | --- | --- | --- | --- | --- | --- | --- | --- |
| Vmean | fixed | Mesurment | 0.1008289 | 0.1278712 | 0.7885192 | 46.61363 | 0.4343851 | -0.1564710 | 0.3581288 |
| ENTR | fixed | Mesurment | 0.0909080 | 0.1266048 | 0.7180454 | 45.68250 | 0.4763856 | -0.1639820 | 0.3457979 |
| Lmean | fixed | Mesurment | 0.0801385 | 0.1275660 | 0.6282124 | 45.96805 | 0.5329737 | -0.1766433 | 0.3369204 |
| LAM | fixed | Mesurment | 0.0679259 | 0.1285140 | 0.5285485 | 48.32561 | 0.5995378 | -0.1904238 | 0.3262756 |
| DET | fixed | Mesurment | 0.0655077 | 0.1291957 | 0.5070425 | 46.31448 | 0.6145308 | -0.1945020 | 0.3255174 |
| Dim.1 | fixed | Mesurment | 0.0667570 | 0.1329279 | 0.5022046 | 50.34731 | 0.6177130 | -0.2001909 | 0.3337049 |
| TREND | fixed | Mesurment | -0.0284925 | 0.1365526 | -0.2086554 | 39.22913 | 0.8357969 | -0.3046447 | 0.2476598 |
| DIV | fixed | Mesurment | 0.0250773 | 0.1436663 | 0.1745523 | 49.61454 | 0.8621418 | -0.2635405 | 0.3136950 |
| REC | fixed | Mesurment | 0.0178624 | 0.1312849 | 0.1360585 | 49.85424 | 0.8923230 | -0.2458501 | 0.2815750 |

## Metrics ~ setting models

##### Formula:Mesurment ~ setting + Age + Sex Number of observations:57

| MesurmentName | term | estimate | std.error | statistic | p.value | conf.low | conf.high |
| --- | --- | --- | --- | --- | --- | --- | --- |
| Lmean | settingMountainous | 0.8409589 | 0.2328783 | 3.6111517 | 0.0006770 | 0.3845258 | 1.2973920 |
| Vmean | settingMountainous | 0.8331149 | 0.2311302 | 3.6045268 | 0.0006910 | 0.3801081 | 1.2861217 |
| DET | settingMountainous | 0.7767704 | 0.2302093 | 3.3741919 | 0.0013907 | 0.3255684 | 1.2279723 |
| Dim.1 | settingMountainous | 0.7454419 | 0.2240595 | 3.3269811 | 0.0016003 | 0.3062932 | 1.1845905 |
| LAM | settingMountainous | 0.7642029 | 0.2312313 | 3.3049287 | 0.0017082 | 0.3109979 | 1.2174079 |
| DIV | settingMountainous | -0.6525598 | 0.2112233 | -3.0894315 | 0.0031910 | -1.0665498 | -0.2385698 |
| ENTR | settingMountainous | 0.7075477 | 0.2423515 | 2.9195102 | 0.0051372 | 0.2325475 | 1.1825479 |
| TREND | settingMountainous | -0.3223264 | 0.2361081 | -1.3651646 | 0.1779684 | -0.7850897 | 0.1404369 |
| REC | settingMountainous | 0.1808659 | 0.2528558 | 0.7152928 | 0.4775675 | -0.3147223 | 0.6764541 |


RQA sensitivity analysis skin temperature(radius = 0.01)


## RQA sensitivity analysis skin temperature(radius = 0.01)

# RQA sensitivity analysis skin temperature(radius = 0.01)

#### Nikolaos Efthymiou

#### 16/11/2022

**Participants:**- We have 106 temperature measurements
- 53 for skin and 53 for air temperature
- In those 53 skin temperature files we have a total of 37 participants
- We drop 21 participants because they dont have temperature measurements
  in both settings (16 participants left)
- We drop 1 participant because of invalid biomarker measurements (15
  participants left)
- We drop 1 participant because of faulty skin temperature measurements
  (14 participants left)

|  | First urban (N=8) | First mountainous (N=6) | Overall (N=14) | P-value |
| --- | --- | --- | --- | --- |
| Age |  |  |  |  |
| Mean (SD) | 42.0 (7.80) | 45.3 (10.9) | 43.4 (9.01) | 0.54 |
| Median [Min, Max] | 39.5 [32.0, 56.0] | 44.5 [31.0, 60.0] | 40.5 [31.0, 60.0] |  |
| Sex |  |  |  |  |
| Female | 6 (75.0%) | 5 (83.3%) | 11 (78.6%) | 1 |
| Male | 2 (25.0%) | 1 (16.7%) | 3 (21.4%) |  |
| BMI |  |  |  |  |
| Mean (SD) | 25.9 (3.67) | 23.7 (2.19) | 24.9 (3.22) | 0.186 |
| Median [Min, Max] | 26.0 [19.4, 29.8] | 24.0 [21.0, 27.2] | 24.6 [19.4, 29.8] |  |
| BMIcat |  |  |  |  |
| underweight | 0 (0%) | 0 (0%) | 0 (0%) | 0.301 |
| normal weight | 4 (50.0%) | 5 (83.3%) | 9 (64.3%) |  |
| overweight | 4 (50.0%) | 1 (16.7%) | 5 (35.7%) |  |
| education\_level |  |  |  |  |
| Secondary | 3 (37.5%) | 2 (33.3%) | 5 (35.7%) | 0.199 |
| University/college | 3 (37.5%) | 0 (0%) | 3 (21.4%) |  |
| Master/PhD | 2 (25.0%) | 4 (66.7%) | 6 (42.9%) |  |
| Smoking\_status |  |  |  |  |
| Smoker | 2 (25.0%) | 0 (0%) | 2 (14.3%) | 0.473 |
| Non-smoker | 5 (62.5%) | 6 (100%) | 11 (78.6%) |  |
| Former smoker | 1 (12.5%) | 0 (0%) | 1 (7.1%) |  |
| alcohol\_freq |  |  |  |  |
| Weekly | 2 (25.0%) | 1 (16.7%) | 3 (21.4%) | 1 |
| Monthly | 2 (25.0%) | 1 (16.7%) | 3 (21.4%) |  |
| Rarely/Never | 4 (50.0%) | 4 (66.7%) | 8 (57.1%) |  |
| Physical\_exercise |  |  |  |  |
| Yes | 1 (12.5%) | 6 (100%) | 7 (50.0%) | 0.00466 |
| No | 7 (87.5%) | 0 (0%) | 7 (50.0%) |  |
| screen\_hours\_day |  |  |  |  |
| Mean (SD) | 5.88 (3.48) | 4.67 (3.08) | 5.36 (3.25) | 0.506 |
| Median [Min, Max] | 6.50 [2.00, 10.0] | 4.00 [1.00, 10.0] | 5.00 [1.00, 10.0] |  |
| days\_mountain |  |  |  |  |
| Mean (SD) | 7.00 (1.20) | 8.67 (4.59) | 7.71 (3.10) | 0.422 |
| Median [Min, Max] | 7.50 [5.00, 8.00] | 6.50 [5.00, 15.0] | 7.00 [5.00, 15.0] |  |
| washout\_days |  |  |  |  |
| Mean (SD) | 11.9 (7.28) | 19.7 (8.41) | 15.2 (8.47) | 0.0995 |
| Median [Min, Max] | 14.0 [2.00, 22.0] | 22.5 [6.00, 30.0] | 15.0 [2.00, 30.0] |  |

RQA analysis Settings: time.lag=1, radius = 0.01


| ID | setting | REC | DET | DIV | Lmean | ENTR | TREND | LAM | Vmean |
| --- | --- | --- | --- | --- | --- | --- | --- | --- | --- |
| T10-U | Urban | 0.0031105 | 0.6651163 | 0.0833333 | 4.409044 | 1.3156262 | -4.2e-06 | 0.4072868 | 3.507343 |
| T11-U | Urban | 0.0028684 | 0.5995293 | 0.1111111 | 4.375460 | 1.0698304 | 0.0e+00 | 0.3873571 | 2.935032 |
| T12-U | Urban | 0.0031626 | 0.6160415 | 0.1000000 | 4.060301 | 1.0494662 | -4.0e-06 | 0.3833486 | 3.099877 |
| T16-U | Urban | 0.0025868 | 0.7166294 | 0.1250000 | 4.433679 | 1.2128118 | -3.6e-06 | 0.5460477 | 3.115957 |
| T17-U | Urban | 0.0033835 | 0.5843786 | 0.1250000 | 3.857009 | 0.9536722 | -2.2e-06 | 0.3514823 | 2.837744 |
| T24-U | Urban | 0.0033397 | 0.5426523 | 0.2000000 | 5.984190 | 0.8209181 | -2.4e-06 | 0.1770609 | 2.806818 |
| T26-U | Urban | 0.0012625 | 0.7028266 | 0.2000000 | 12.026144 | 1.0899208 | -3.0e-07 | 0.1436211 | 2.724638 |
| T29-U | Urban | 0.0025251 | 0.6734148 | 0.0909091 | 4.435220 | 1.0759797 | -2.0e-06 | 0.4027884 | 3.038905 |
| T31-U | Urban | 0.0019261 | 0.6424637 | 0.1250000 | 5.614880 | 0.9128312 | -1.0e-06 | 0.2999499 | 2.845606 |
| T33-U | Urban | 0.0020390 | 0.6144749 | 0.1666667 | 5.423800 | 0.8561531 | -1.7e-06 | 0.2554399 | 2.634146 |
| T34-U | Urban | 0.0017380 | 0.6625971 | 0.1428571 | 6.506812 | 1.0551371 | -1.0e-07 | 0.2228080 | 2.909420 |
| T35-U | Urban | 0.0052141 | 0.6054384 | 0.0833333 | 3.331298 | 1.0523146 | -6.9e-06 | 0.4513504 | 2.929172 |
| T45-U | Urban | 0.0020949 | 0.6091160 | 0.1111111 | 5.478261 | 0.9497143 | 5.0e-07 | 0.2520718 | 2.904509 |
| T60-U | Urban | 0.0024826 | 0.6588967 | 0.0909091 | 4.652949 | 1.1260586 | -1.4e-06 | 0.4015152 | 3.080477 |
| T10-M | Mountainous | 0.0051331 | 0.6899662 | 0.1250000 | 3.420587 | 1.1944846 | -8.0e-06 | 0.5641676 | 3.408059 |
| T11-M | Mountainous | 0.0027537 | 0.6322242 | 0.0909091 | 4.575412 | 1.1644048 | -3.6e-06 | 0.4021016 | 3.489362 |
| T12-M | Mountainous | 0.0053221 | 0.7432041 | 0.0476190 | 3.987360 | 1.5402978 | -8.2e-06 | 0.5940558 | 3.622099 |
| T16-M | Mountainous | 0.0041011 | 0.6380527 | 0.1000000 | 3.581518 | 1.0861061 | -4.1e-06 | 0.4965898 | 3.194402 |
| T17-M | Mountainous | 0.0028810 | 0.5775025 | 0.1000000 | 4.296388 | 0.9333495 | -2.3e-06 | 0.3093405 | 3.074875 |
| T24-M | Mountainous | 0.0018943 | 0.6471487 | 0.1000000 | 6.184915 | 1.1303381 | -1.0e-06 | 0.3187373 | 3.068627 |
| T26-M | Mountainous | 0.0029205 | 0.6786658 | 0.0833333 | 4.551495 | 1.3179160 | -3.3e-06 | 0.4686262 | 3.370546 |
| T29-M | Mountainous | 0.0019917 | 0.6348668 | 0.1428571 | 5.451143 | 0.9146337 | -2.1e-06 | 0.2859564 | 2.908867 |
| T31-M | Mountainous | 0.0019290 | 0.7075000 | 0.1111111 | 5.431862 | 1.1153709 | -2.3e-06 | 0.3700000 | 3.325843 |
| T33-M | Mountainous | 0.0035523 | 0.6994298 | 0.1000000 | 4.158192 | 1.3804703 | -5.2e-06 | 0.5585121 | 3.602452 |
| T34-M | Mountainous | 0.0047877 | 0.6866256 | 0.0769231 | 4.453721 | 1.2829722 | -8.8e-06 | 0.4658646 | 3.611714 |
| T35-M | Mountainous | 0.0030064 | 0.7115816 | 0.1111111 | 4.073462 | 1.1974539 | -4.7e-06 | 0.4469041 | 3.744624 |
| T45-M | Mountainous | 0.0034664 | 0.6947691 | 0.0833333 | 3.998399 | 1.2623905 | 4.0e-07 | 0.4993044 | 3.221723 |
| T60-M | Mountainous | 0.0031636 | 0.6216463 | 0.1000000 | 3.917387 | 0.9913849 | 6.4e-06 | 0.4243902 | 3.121076 |

## RQA Plots

## PCA analysis

Eigenvalues/variances

|  | eigenvalue | variance.percent | cumulative.variance.percent |
| --- | --- | --- | --- |
| Dim.1 | 4.7494601 | 59.368251 | 59.36825 |
| Dim.2 | 1.5054120 | 18.817650 | 78.18590 |
| Dim.3 | 0.8528720 | 10.660900 | 88.84680 |
| Dim.4 | 0.3132352 | 3.915440 | 92.76224 |
| Dim.5 | 0.2533579 | 3.166973 | 95.92921 |
| Dim.6 | 0.2122167 | 2.652709 | 98.58192 |
| Dim.7 | 0.0807063 | 1.008829 | 99.59075 |
| Dim.8 | 0.0327398 | 0.409247 | 100.00000 |

Coordinates of variables

|  | Dim.1 | Dim.2 | Dim.3 | Dim.4 | Dim.5 |
| --- | --- | --- | --- | --- | --- |
| REC | 0.7430280 | -0.4359079 | 0.3660445 | 0.1772369 | 0.2084344 |
| DET | 0.5820278 | 0.7572932 | -0.0493805 | 0.2009271 | -0.0692015 |
| DIV | -0.7953843 | 0.1811620 | 0.4322483 | 0.2352746 | -0.2506783 |
| Lmean | -0.6424312 | 0.6780960 | 0.2318447 | -0.0522034 | 0.2316912 |
| ENTR | 0.8533579 | 0.4439616 | -0.0449985 | -0.0176829 | 0.1637956 |
| TREND | -0.6757766 | 0.0250364 | -0.6744289 | 0.1853040 | 0.0592626 |
| LAM | 0.9313287 | -0.0877648 | -0.1239588 | 0.2884090 | -0.0672563 |
| Vmean | 0.8723487 | 0.2093754 | -0.0600789 | -0.2560102 | -0.2318173 |

Contribution of variables

|  | Dim.1 | Dim.2 | Dim.3 | Dim.4 | Dim.5 |
| --- | --- | --- | --- | --- | --- |
| REC | 11.624282 | 12.6221730 | 15.7102824 | 10.0285466 | 17.147638 |
| DET | 7.132523 | 38.0954211 | 0.2859089 | 12.8886188 | 1.890152 |
| DIV | 13.320172 | 2.1801130 | 21.9069901 | 17.6717484 | 24.802701 |
| Lmean | 8.689785 | 30.5440719 | 6.3024655 | 0.8700170 | 21.187744 |
| ENTR | 15.332684 | 13.0928870 | 0.2374168 | 0.0998245 | 10.589362 |
| TREND | 9.615281 | 0.0416379 | 53.3320702 | 10.9622305 | 1.386202 |
| LAM | 18.262562 | 0.5116651 | 1.8016524 | 26.5550446 | 1.785384 |
| Vmean | 16.022710 | 2.9120310 | 0.4232136 | 20.9239697 | 21.210817 |

## RQA descriptives Overall and by setting

| metric | setting | mean | sd | q0 | q25 | q50 | q75 | q90 | q95 | q100 |
| --- | --- | --- | --- | --- | --- | --- | --- | --- | --- | --- |
| DET | Mountainous | 0.6687988 | 0.0445505 | 0.5775025 | 0.6356633 | 0.6826457 | 0.6982646 | 0.7103572 | 0.7226495 | 0.7432041 |
| DET | Overall | 0.6520271 | 0.0484520 | 0.5426523 | 0.6156498 | 0.6530227 | 0.6911669 | 0.7087245 | 0.7148627 | 0.7432041 |
| DET | Urban | 0.6352554 | 0.0478035 | 0.5426523 | 0.6063578 | 0.6292526 | 0.6644865 | 0.6940031 | 0.7076576 | 0.7166294 |
| Dim.1 | Mountainous | 1.1212836 | 1.9942329 | -2.1011367 | -0.6954468 | 1.2971772 | 2.5419653 | 3.0602009 | 3.8313149 | 5.0861693 |
| Dim.1 | Overall | 0.0000000 | 2.2193166 | -4.3192438 | -1.4117562 | 0.0135987 | 1.4699917 | 2.7765368 | 3.0442970 | 5.0861693 |
| Dim.1 | Urban | -1.1212836 | 1.8827269 | -4.3192438 | -2.2833950 | -1.0498720 | 0.0216294 | 1.1894108 | 1.4084413 | 1.6928641 |
| Dim.2 | Mountainous | 0.1221144 | 0.8977582 | -1.5480915 | -0.3627101 | 0.2154565 | 0.8476908 | 0.9553766 | 1.1322241 | 1.4037162 |
| Dim.2 | Overall | 0.0000000 | 1.2494671 | -2.3118214 | -1.0001806 | 0.0372260 | 0.7949278 | 1.0339460 | 1.3072466 | 3.9821538 |
| Dim.2 | Urban | -0.1221144 | 1.5505928 | -2.3118214 | -1.0076567 | -0.2061423 | 0.3947084 | 1.0877419 | 2.1270116 | 3.9821538 |
| DIV | Mountainous | 0.0980141 | 0.0224772 | 0.0476190 | 0.0852273 | 0.1000000 | 0.1083333 | 0.1208333 | 0.1312500 | 0.1428571 |
| DIV | Overall | 0.1116939 | 0.0343825 | 0.0476190 | 0.0909091 | 0.1000000 | 0.1250000 | 0.1500000 | 0.1883333 | 0.2000000 |
| DIV | Urban | 0.1253736 | 0.0393314 | 0.0833333 | 0.0931818 | 0.1180556 | 0.1383929 | 0.1900000 | 0.2000000 | 0.2000000 |
| ENTR | Mountainous | 1.1793981 | 0.1730848 | 0.9146337 | 1.0934223 | 1.1794447 | 1.2778268 | 1.3617040 | 1.4364099 | 1.5402978 |
| ENTR | Overall | 1.1090003 | 0.1677911 | 0.8209181 | 0.9819567 | 1.0880135 | 1.2012934 | 1.3163131 | 1.3585763 | 1.5402978 |
| ENTR | Urban | 1.0386024 | 0.1335699 | 0.8209181 | 0.9507038 | 1.0537258 | 1.0864355 | 1.1867858 | 1.2487968 | 1.3156262 |
| LAM | Mountainous | 0.4431822 | 0.0974267 | 0.2859564 | 0.3780254 | 0.4563843 | 0.4986258 | 0.5624709 | 0.5746285 | 0.5940558 |
| LAM | Overall | 0.3888100 | 0.1174475 | 0.1436211 | 0.3069928 | 0.4018084 | 0.4665550 | 0.5497870 | 0.5621882 | 0.5940558 |
| LAM | Urban | 0.3344377 | 0.1130904 | 0.1436211 | 0.2529138 | 0.3674155 | 0.4024701 | 0.4381313 | 0.4844944 | 0.5460477 |
| Lmean | Mountainous | 4.4344172 | 0.7728594 | 3.4205869 | 3.9901199 | 4.2272903 | 4.5694327 | 5.4453590 | 5.7079634 | 6.1849148 |
| Lmean | Overall | 4.8811031 | 1.6297384 | 3.3312977 | 4.0448258 | 4.4344497 | 5.4366822 | 6.0444073 | 6.3941480 | 12.0261438 |
| Lmean | Urban | 5.3277890 | 2.1188065 | 3.3312977 | 4.3838561 | 4.5440847 | 5.5807250 | 6.3500253 | 8.4385781 | 12.0261438 |
| REC | Mountainous | 0.0033502 | 0.0011317 | 0.0018943 | 0.0027855 | 0.0030850 | 0.0039639 | 0.0050295 | 0.0051993 | 0.0053221 |
| REC | Overall | 0.0030227 | 0.0010843 | 0.0012625 | 0.0020809 | 0.0029008 | 0.0034042 | 0.0048913 | 0.0051858 | 0.0053221 |
| REC | Urban | 0.0026953 | 0.0009645 | 0.0012625 | 0.0020530 | 0.0025559 | 0.0031496 | 0.0033704 | 0.0040242 | 0.0052141 |
| TREND | Mountainous | -0.0000033 | 0.0000039 | -0.0000088 | -0.0000051 | -0.0000035 | -0.0000021 | 0.0000000 | 0.0000025 | 0.0000064 |
| TREND | Overall | -0.0000027 | 0.0000031 | -0.0000088 | -0.0000041 | -0.0000023 | -0.0000010 | 0.0000001 | 0.0000005 | 0.0000064 |
| TREND | Urban | -0.0000021 | 0.0000020 | -0.0000069 | -0.0000033 | -0.0000019 | -0.0000005 | 0.0000000 | 0.0000002 | 0.0000005 |
| Vmean | Mountainous | 3.3403050 | 0.2522222 | 2.9088670 | 3.1394078 | 3.3481945 | 3.5741793 | 3.6189837 | 3.6649829 | 3.7446237 |
| Vmean | Overall | 3.1476398 | 0.3012633 | 2.6341463 | 2.9092820 | 3.0901768 | 3.3799245 | 3.6052304 | 3.6184644 | 3.7446237 |
| Vmean | Urban | 2.9549746 | 0.2119768 | 2.6341463 | 2.8397098 | 2.9192960 | 3.0700839 | 3.1111332 | 3.2529424 | 3.5073431 |

## Paired T test after log transformation for each variable for the 2 groups (alternative hypothesis: true difference in means is not equal to 0)

Paired - T test


| metric | estimate | statistic | p.value | parameter | conf.low | conf.high | method | alternative |
| --- | --- | --- | --- | --- | --- | --- | --- | --- |
| REC | -0.0006528 | -1.634197 | 0.1261890 | 13 | -0.0015157 | 0.0002102 | Paired t-test | two.sided |
| DET | -0.0203695 | -1.969764 | 0.0705507 | 13 | -0.0427100 | 0.0019711 | Paired t-test | two.sided |
| DIV | 0.0242502 | 2.044181 | 0.0617409 | 13 | -0.0013783 | 0.0498787 | Paired t-test | two.sided |
| Lmean | 0.1231823 | 1.764756 | 0.1010702 | 13 | -0.0276143 | 0.2739789 | Paired t-test | two.sided |
| ENTR | -0.0658575 | -2.294793 | 0.0390341 | 13 | -0.1278573 | -0.0038578 | Paired t-test | two.sided |
| TREND | 0.0000012 | 1.211281 | 0.2473472 | 13 | -0.0000010 | 0.0000035 | Paired t-test | two.sided |
| LAM | -0.0795726 | -2.839081 | 0.0139458 | 13 | -0.1401226 | -0.0190227 | Paired t-test | two.sided |
| Vmean | -0.0926890 | -4.271823 | 0.0009096 | 13 | -0.1395641 | -0.0458138 | Paired t-test | two.sided |
| Dim.1 | -0.5047140 | -2.884454 | 0.0127837 | 13 | -0.8827295 | -0.1266985 | Paired t-test | two.sided |

## Leptin,cortisol and adiponectin are adjusted for creatinine and then log transformed (Metrics are log transformed in a previous step)

## Leptin models

##### Formula:leptin ~ Mesurment + SampleType + Age + (1 | ID) Number of observations:56

p.values values for Metabolites (14 total models)


| MesurmentName | effect | term | estimate | std.error | statistic | df | p.value | conf.low | conf.high |
| --- | --- | --- | --- | --- | --- | --- | --- | --- | --- |
| Lmean | fixed | Mesurment | -0.2088080 | 0.1372291 | -1.5216018 | 49.29715 | 0.1344980 | -0.4845382 | 0.0669221 |
| DIV | fixed | Mesurment | -0.1341712 | 0.1134301 | -1.1828530 | 45.80048 | 0.2429714 | -0.3625211 | 0.0941787 |
| TREND | fixed | Mesurment | 0.1474665 | 0.1356959 | 1.0867426 | 51.91942 | 0.2821703 | -0.1248373 | 0.4197703 |
| DET | fixed | Mesurment | -0.1185685 | 0.1208984 | -0.9807285 | 50.95352 | 0.3313605 | -0.3612876 | 0.1241505 |
| Dim.1 | fixed | Mesurment | 0.0652513 | 0.1197252 | 0.5450089 | 45.53865 | 0.5884068 | -0.1758090 | 0.3063116 |
| Vmean | fixed | Mesurment | -0.0383927 | 0.1107958 | -0.3465181 | 45.13261 | 0.7305638 | -0.2615287 | 0.1847433 |
| LAM | fixed | Mesurment | 0.0406760 | 0.1224171 | 0.3322740 | 47.60100 | 0.7411412 | -0.2055133 | 0.2868653 |
| ENTR | fixed | Mesurment | 0.0200383 | 0.1169888 | 0.1712842 | 48.56722 | 0.8647120 | -0.2151124 | 0.2551891 |
| REC | fixed | Mesurment | 0.0069768 | 0.1237264 | 0.0563888 | 48.98788 | 0.9552615 | -0.2416622 | 0.2556158 |

## Adiponectin models

##### Formula:adiponectin ~ Mesurment + SampleType + Age + (1 | ID) Number of observations:56

p.values values for Metabolites (14 total models)


| MesurmentName | effect | term | estimate | std.error | statistic | df | p.value | conf.low | conf.high |
| --- | --- | --- | --- | --- | --- | --- | --- | --- | --- |
| Lmean | fixed | Mesurment | -0.3142429 | 0.1408483 | -2.2310742 | 50.71038 | 0.0301264 | -0.5970468 | -0.0314390 |
| DIV | fixed | Mesurment | -0.2202148 | 0.1174042 | -1.8756980 | 46.71292 | 0.0669541 | -0.4564398 | 0.0160103 |
| REC | fixed | Mesurment | 0.1997830 | 0.1269095 | 1.5742169 | 49.98873 | 0.1217458 | -0.0551236 | 0.4546896 |
| Dim.1 | fixed | Mesurment | 0.1661310 | 0.1246517 | 1.3327610 | 46.42925 | 0.1891140 | -0.0847174 | 0.4169793 |
| LAM | fixed | Mesurment | 0.1634453 | 0.1270541 | 1.2864232 | 48.78264 | 0.2043673 | -0.0919082 | 0.4187988 |
| DET | fixed | Mesurment | -0.1021333 | 0.1269768 | -0.8043466 | 51.91558 | 0.4248649 | -0.3569408 | 0.1526741 |
| ENTR | fixed | Mesurment | 0.0853971 | 0.1224216 | 0.6975662 | 49.54486 | 0.4887093 | -0.1605498 | 0.3313441 |
| Vmean | fixed | Mesurment | 0.0741134 | 0.1166492 | 0.6353530 | 45.82808 | 0.5283581 | -0.1607130 | 0.3089398 |
| TREND | fixed | Mesurment | 0.0786865 | 0.1423683 | 0.5526969 | 51.69590 | 0.5828531 | -0.2070364 | 0.3644095 |

## Cortisol models

##### Formula:cortisol ~ Mesurment + SampleType + Age + (1 | ID) Number of observations:56

p.values values for Metabolites (14 total models)


| MesurmentName | effect | term | estimate | std.error | statistic | df | p.value | conf.low | conf.high |
| --- | --- | --- | --- | --- | --- | --- | --- | --- | --- |
| DIV | fixed | Mesurment | 0.1924551 | 0.1200615 | 1.6029704 | 51.63207 | 0.1150419 | -0.0485069 | 0.4334170 |
| ENTR | fixed | Mesurment | -0.1139453 | 0.1202555 | -0.9475266 | 51.86215 | 0.3477648 | -0.3552708 | 0.1273803 |
| Vmean | fixed | Mesurment | -0.0838622 | 0.1184650 | -0.7079069 | 50.48114 | 0.4822589 | -0.3217499 | 0.1540256 |
| Dim.1 | fixed | Mesurment | -0.0787399 | 0.1279281 | -0.6155012 | 50.84922 | 0.5409690 | -0.3355847 | 0.1781050 |
| TREND | fixed | Mesurment | 0.0563030 | 0.1343897 | 0.4189531 | 44.21054 | 0.6772790 | -0.2145053 | 0.3271113 |
| DET | fixed | Mesurment | -0.0463371 | 0.1226586 | -0.3777726 | 48.84782 | 0.7072361 | -0.2928482 | 0.2001740 |
| Lmean | fixed | Mesurment | -0.0443880 | 0.1438255 | -0.3086242 | 51.46723 | 0.7588532 | -0.3330660 | 0.2442900 |
| LAM | fixed | Mesurment | 0.0088958 | 0.1282390 | 0.0693688 | 51.99369 | 0.9449621 | -0.2484354 | 0.2662270 |
| REC | fixed | Mesurment | -0.0011286 | 0.1274324 | -0.0088562 | 51.32678 | 0.9929682 | -0.2569202 | 0.2546630 |

## Metrics ~ setting models

##### Formula:Mesurment ~ setting + Age + Sex Number of observations:57

| MesurmentName | term | estimate | std.error | statistic | p.value | conf.low | conf.high |
| --- | --- | --- | --- | --- | --- | --- | --- |
| Vmean | settingMountainous | 1.2508206 | 0.2040879 | 6.128834 | 0.0000001 | 0.8508158 | 1.6508255 |
| Dim.1 | settingMountainous | 0.9285503 | 0.2144415 | 4.330087 | 0.0000666 | 0.5082527 | 1.3488478 |
| LAM | settingMountainous | 0.8821916 | 0.2229937 | 3.956128 | 0.0002277 | 0.4451319 | 1.3192512 |
| DIV | settingMountainous | -0.7509147 | 0.2382505 | -3.151786 | 0.0026695 | -1.2178771 | -0.2839523 |
| ENTR | settingMountainous | 0.7851442 | 0.2491699 | 3.151039 | 0.0026752 | 0.2967801 | 1.2735082 |
| DET | settingMountainous | 0.6797367 | 0.2556364 | 2.658998 | 0.0103411 | 0.1786986 | 1.1807747 |
| Lmean | settingMountainous | -0.5369285 | 0.2161210 | -2.484388 | 0.0161713 | -0.9605179 | -0.1133391 |
| REC | settingMountainous | 0.5601171 | 0.2481667 | 2.257020 | 0.0281535 | 0.0737194 | 1.0465148 |
| TREND | settingMountainous | -0.3839562 | 0.2450466 | -1.566870 | 0.1230968 | -0.8642386 | 0.0963263 |


RQA analysis personal air temperature


## RQA analysis personal air temperature

# RQA analysis personal air temperature

#### Nikolaos Efthymiou

#### 16/11/2022

**Participants:**- We have 106 temperature measurements
- 53 for skin and 53 for air temperature
- In those 53 skin temperature files we have a total of 37 participants
- We drop 21 participants because they dont have temperature measurements
  in both settings (16 participants left)
- We drop 1 participant because of invalid biomarker measurements (15
  participants left)

|  | First urban (N=6) | First mountainous (N=9) | Overall (N=15) | P-value |
| --- | --- | --- | --- | --- |
| Age |  |  |  |  |
| Mean (SD) | 40.0 (8.29) | 43.1 (11.2) | 41.9 (9.93) | 0.548 |
| Median [Min, Max] | 38.0 [32.0, 56.0] | 40.0 [29.0, 60.0] | 38.0 [29.0, 60.0] |  |
| Sex |  |  |  |  |
| Female | 5 (83.3%) | 5 (55.6%) | 10 (66.7%) | 0.58 |
| Male | 1 (16.7%) | 4 (44.4%) | 5 (33.3%) |  |
| BMI |  |  |  |  |
| Mean (SD) | 24.8 (3.70) | 24.3 (2.37) | 24.5 (2.86) | 0.753 |
| Median [Min, Max] | 25.0 [19.4, 30.1] | 24.1 [21.0, 28.1] | 24.2 [19.4, 30.1] |  |
| BMIcat |  |  |  |  |
| underweight | 0 (0%) | 0 (0%) | 0 (0%) | 1 |
| normal weight | 4 (66.7%) | 6 (66.7%) | 10 (66.7%) |  |
| overweight | 2 (33.3%) | 3 (33.3%) | 5 (33.3%) |  |
| education\_level |  |  |  |  |
| Secondary | 2 (33.3%) | 2 (22.2%) | 4 (26.7%) | 0.664 |
| University/college | 2 (33.3%) | 2 (22.2%) | 4 (26.7%) |  |
| Master/PhD | 2 (33.3%) | 5 (55.6%) | 7 (46.7%) |  |
| Smoking\_status |  |  |  |  |
| Smoker | 1 (16.7%) | 1 (11.1%) | 2 (13.3%) | 1 |
| Non-smoker | 4 (66.7%) | 7 (77.8%) | 11 (73.3%) |  |
| Former smoker | 1 (16.7%) | 1 (11.1%) | 2 (13.3%) |  |
| alcohol\_freq |  |  |  |  |
| Weekly | 2 (33.3%) | 3 (33.3%) | 5 (33.3%) | 1 |
| Monthly | 1 (16.7%) | 2 (22.2%) | 3 (20.0%) |  |
| Rarely/Never | 3 (50.0%) | 4 (44.4%) | 7 (46.7%) |  |
| Physical\_exercise |  |  |  |  |
| Yes | 1 (16.7%) | 7 (77.8%) | 8 (53.3%) | 0.0406 |
| No | 5 (83.3%) | 2 (22.2%) | 7 (46.7%) |  |
| screen\_hours\_day |  |  |  |  |
| Mean (SD) | 6.67 (3.72) | 5.06 (4.38) | 5.70 (4.07) | 0.459 |
| Median [Min, Max] | 8.00 [2.00, 10.0] | 4.00 [0.500, 14.0] | 4.00 [0.500, 14.0] |  |
| days\_mountain |  |  |  |  |
| Mean (SD) | 7.00 (1.26) | 8.78 (4.47) | 8.07 (3.58) | 0.286 |
| Median [Min, Max] | 7.50 [5.00, 8.00] | 6.00 [5.00, 15.0] | 7.00 [5.00, 15.0] |  |
| washout\_days |  |  |  |  |
| Mean (SD) | 14.3 (8.87) | 15.8 (9.22) | 15.2 (8.78) | 0.767 |
| Median [Min, Max] | 15.0 [2.00, 28.0] | 14.0 [5.00, 30.0] | 15.0 [2.00, 30.0] |  |

RQA analysis Settings: time.lag=1, radius = 0.1


| ID | setting | REC | DET | DIV | Lmean | ENTR | TREND | LAM | Vmean |
| --- | --- | --- | --- | --- | --- | --- | --- | --- | --- |
| T10-U | Urban | 0.0906154 | 0.9867376 | 0.0015528 | 18.229083 | 3.214731 | -0.0001261 | 0.9871953 | 23.137583 |
| T11-U | Urban | 0.0620824 | 0.9384933 | 0.0023095 | 15.531045 | 2.677008 | -0.0000421 | 0.9392701 | 21.877329 |
| T12-U | Urban | 0.0938927 | 0.9635432 | 0.0024510 | 31.747842 | 2.645197 | -0.0001185 | 0.9669382 | 39.171660 |
| T16-U | Urban | 0.0354417 | 0.9132423 | 0.0025510 | 8.874256 | 2.460251 | -0.0000030 | 0.9211071 | 11.469671 |
| T17-U | Urban | 0.1965548 | 0.9915893 | 0.0006949 | 22.209595 | 3.564735 | -0.0001608 | 0.9896436 | 31.003459 |
| T18-U | Urban | 0.0627913 | 0.9736414 | 0.0031153 | 8.925720 | 2.756333 | -0.0001199 | 0.9766213 | 10.373634 |
| T19-U | Urban | 0.0737664 | 0.9784129 | 0.0023256 | 19.273664 | 3.513661 | 0.0000748 | 0.9809691 | 26.027927 |
| T24-U | Urban | 0.0258567 | 0.9103406 | 0.0028986 | 7.706114 | 2.328397 | -0.0000744 | 0.9129503 | 7.587529 |
| T26-U | Urban | 0.0093528 | 0.7821036 | 0.0117647 | 5.030555 | 1.912337 | -0.0000012 | 0.7445139 | 5.505536 |
| T29-U | Urban | 0.0395120 | 0.9669238 | 0.0022624 | 12.082050 | 2.724401 | -0.0000115 | 0.9765171 | 15.599142 |
| T35-U | Urban | 0.0297049 | 0.9164556 | 0.0023697 | 6.838280 | 2.218178 | -0.0000177 | 0.9463114 | 7.917550 |
| T37-U | Urban | 0.0480623 | 0.9362244 | 0.0021097 | 10.068631 | 2.700193 | -0.0000022 | 0.9490678 | 14.370404 |
| T42-U | Urban | 0.2210050 | 0.9892117 | 0.0006949 | 40.156967 | 3.920029 | -0.0003904 | 0.9888561 | 51.484776 |
| T45-U | Urban | 0.1151100 | 0.9841050 | 0.0011737 | 19.089638 | 3.319851 | -0.0001741 | 0.9895095 | 21.171388 |
| T60-U | Urban | 0.0560407 | 0.9840112 | 0.0013459 | 15.215968 | 3.412501 | -0.0000867 | 0.9831936 | 20.552797 |
| T10-M | Mountainous | 0.1105295 | 0.9887955 | 0.0009183 | 25.500844 | 3.925029 | -0.0001841 | 0.9866576 | 33.338641 |
| T11-M | Mountainous | 0.0336169 | 0.9230791 | 0.0021598 | 14.508681 | 2.872387 | -0.0000173 | 0.9311844 | 19.954196 |
| T12-M | Mountainous | 0.0170968 | 0.7758095 | 0.0149254 | 4.127870 | 1.713220 | -0.0000123 | 0.7655421 | 4.290231 |
| T16-M | Mountainous | 0.0358738 | 0.9760445 | 0.0020000 | 12.143502 | 2.743602 | -0.0000479 | 0.9819729 | 15.499045 |
| T17-M | Mountainous | 0.0248910 | 0.9495873 | 0.0018657 | 8.194616 | 2.599844 | -0.0000267 | 0.9517960 | 9.578085 |
| T18-M | Mountainous | 0.0241368 | 0.9182817 | 0.0023364 | 9.447071 | 2.546988 | -0.0000281 | 0.9162837 | 12.027275 |
| T19-M | Mountainous | 0.0139477 | 0.8754581 | 0.0027548 | 7.822057 | 2.117364 | -0.0000201 | 0.8405020 | 7.928571 |
| T24-M | Mountainous | 0.0206491 | 0.9499743 | 0.0025316 | 8.836846 | 2.644220 | -0.0000024 | 0.9611145 | 10.181346 |
| T26-M | Mountainous | 0.0308623 | 0.8883368 | 0.0019763 | 6.988322 | 2.165585 | -0.0000457 | 0.9082599 | 9.073525 |
| T29-M | Mountainous | 0.0795774 | 0.9897200 | 0.0003789 | 33.007574 | 4.112917 | 0.0000081 | 0.9884785 | 42.787535 |
| T35-M | Mountainous | 0.0968094 | 0.9896585 | 0.0016420 | 26.137087 | 3.817988 | 0.0001192 | 0.9879897 | 34.728244 |
| T37-M | Mountainous | 0.0425974 | 0.9208876 | 0.0020704 | 9.865616 | 2.284902 | -0.0000274 | 0.9363523 | 14.128459 |
| T42-M | Mountainous | 0.1130382 | 0.9922183 | 0.0008475 | 19.677807 | 3.761089 | -0.0001835 | 0.9899572 | 26.290732 |
| T45-M | Mountainous | 0.1322695 | 0.9894120 | 0.0018904 | 23.030637 | 3.816057 | -0.0001246 | 0.9861161 | 31.045225 |
| T60-M | Mountainous | 0.0443808 | 0.9708350 | 0.0010246 | 13.871138 | 3.117764 | 0.0001469 | 0.9726714 | 18.683573 |

## RQA Plots

## PCA analysis

Eigenvalues/variances

|  | eigenvalue | variance.percent | cumulative.variance.percent |
| --- | --- | --- | --- |
| Dim.1 | 5.5368563 | 69.2107037 | 69.21070 |
| Dim.2 | 1.3423865 | 16.7798317 | 85.99054 |
| Dim.3 | 0.6789219 | 8.4865233 | 94.47706 |
| Dim.4 | 0.2092409 | 2.6155111 | 97.09257 |
| Dim.5 | 0.1124820 | 1.4060249 | 98.49859 |
| Dim.6 | 0.1042929 | 1.3036619 | 99.80226 |
| Dim.7 | 0.0120282 | 0.1503528 | 99.95261 |
| Dim.8 | 0.0037912 | 0.0473906 | 100.00000 |

Coordinates of variables

|  | Dim.1 | Dim.2 | Dim.3 | Dim.4 | Dim.5 |
| --- | --- | --- | --- | --- | --- |
| REC | 0.8550617 | 0.4169904 | -0.0689137 | 0.1382943 | -0.2563787 |
| DET | 0.9011659 | -0.3912198 | -0.0837199 | 0.1027371 | 0.0703060 |
| DIV | -0.7432210 | 0.5489403 | 0.2311892 | 0.2424873 | 0.1256777 |
| Lmean | 0.8886173 | 0.2975787 | 0.2845661 | -0.1815139 | 0.0661551 |
| ENTR | 0.9171882 | -0.0175699 | 0.2264559 | 0.2398384 | 0.0522549 |
| TREND | -0.5198445 | -0.5869994 | 0.6067968 | 0.0282808 | -0.1175434 |
| LAM | 0.8577871 | -0.4560979 | -0.1346831 | 0.0747107 | 0.0655875 |
| Vmean | 0.8968482 | 0.2695585 | 0.3084044 | -0.1546264 | 0.0280874 |

Contribution of variables

|  | Dim.1 | Dim.2 | Dim.3 | Dim.4 | Dim.5 |
| --- | --- | --- | --- | --- | --- |
| REC | 13.204793 | 12.9531264 | 0.6995056 | 9.1403388 | 58.4360682 |
| DET | 14.667168 | 11.4015532 | 1.0323746 | 5.0443802 | 4.3944173 |
| DIV | 9.976372 | 22.4477374 | 7.8725506 | 28.1016181 | 14.0421354 |
| Lmean | 14.261534 | 6.5966917 | 11.9274188 | 15.7461151 | 3.8908371 |
| ENTR | 15.193353 | 0.0229963 | 7.5534901 | 27.4910161 | 2.4275620 |
| TREND | 4.880717 | 25.6683367 | 54.2333846 | 0.3822402 | 12.2832633 |
| LAM | 13.289107 | 15.4966764 | 2.6718143 | 2.6675870 | 3.8243597 |
| Vmean | 14.526955 | 5.4128819 | 14.0094614 | 11.4267046 | 0.7013569 |

## RQA descriptives Overall and by setting

| metric | setting | mean | sd | q0 | q25 | q50 | q75 | q90 | q95 | q100 |
| --- | --- | --- | --- | --- | --- | --- | --- | --- | --- | --- |
| DET | Mountainous | 0.9398732 | 0.0596688 | 0.7758095 | 0.9195846 | 0.9499743 | 0.9891038 | 0.9896954 | 0.9904695 | 0.9922183 |
| DET | Overall | 0.9437711 | 0.0561871 | 0.7758095 | 0.9189332 | 0.9652335 | 0.9860795 | 0.9896646 | 0.9907481 | 0.9922183 |
| DET | Urban | 0.9476691 | 0.0542823 | 0.7821036 | 0.9263400 | 0.9669238 | 0.9840581 | 0.9882221 | 0.9899250 | 0.9915893 |
| Dim.1 | Mountainous | -0.2223350 | 2.4070502 | -5.6969209 | -1.3007470 | -0.5625083 | 2.1381831 | 2.6716312 | 2.8510664 | 2.9692601 |
| Dim.1 | Overall | 0.0000000 | 2.3932786 | -5.6969209 | -1.3571631 | -0.2009900 | 1.7646034 | 2.8172967 | 2.9827842 | 5.2895124 |
| Dim.1 | Urban | 0.2223350 | 2.4423092 | -5.3262443 | -1.1363372 | -0.1765867 | 1.5650370 | 2.5052882 | 3.6825483 | 5.2895124 |
| Dim.2 | Mountainous | -0.1911245 | 1.1076691 | -1.8502471 | -0.8852321 | -0.3106262 | 0.2263416 | 0.6316591 | 1.3614842 | 2.9658994 |
| Dim.2 | Overall | 0.0000000 | 1.1784209 | -1.8502471 | -0.7805950 | -0.2915102 | 0.3224085 | 1.2188518 | 2.7536407 | 3.2240891 |
| Dim.2 | Urban | 0.1911245 | 1.2535295 | -0.9949641 | -0.6714837 | -0.1936806 | 0.6278377 | 1.9273860 | 2.7131761 | 3.2240891 |
| DIV | Mountainous | 0.0026215 | 0.0034693 | 0.0003789 | 0.0013333 | 0.0019763 | 0.0022481 | 0.0026656 | 0.0064060 | 0.0149254 |
| DIV | Overall | 0.0026314 | 0.0030251 | 0.0003789 | 0.0013976 | 0.0020900 | 0.0024307 | 0.0029202 | 0.0078725 | 0.0149254 |
| DIV | Urban | 0.0026413 | 0.0026305 | 0.0006949 | 0.0014493 | 0.0023095 | 0.0025010 | 0.0030286 | 0.0057101 | 0.0117647 |
| ENTR | Mountainous | 2.9492636 | 0.7653310 | 1.7132196 | 2.4159449 | 2.7436021 | 3.7885725 | 3.8822131 | 3.9813957 | 4.1129167 |
| ENTR | Overall | 2.9202252 | 0.6640469 | 1.7132196 | 2.4819352 | 2.7340018 | 3.4883707 | 3.8281924 | 3.9227793 | 4.1129167 |
| ENTR | Urban | 2.8911868 | 0.5708547 | 1.9123368 | 2.5527241 | 2.7244015 | 3.3661758 | 3.5443054 | 3.6713233 | 3.9200289 |
| LAM | Mountainous | 0.9403252 | 0.0637301 | 0.7655421 | 0.9237340 | 0.9611145 | 0.9863868 | 0.9882830 | 0.9889221 | 0.9899572 |
| LAM | Overall | 0.9452514 | 0.0621278 | 0.7445139 | 0.9324764 | 0.9698048 | 0.9865222 | 0.9889214 | 0.9895833 | 0.9899572 |
| LAM | Urban | 0.9501776 | 0.0623050 | 0.7445139 | 0.9427908 | 0.9765171 | 0.9851945 | 0.9892481 | 0.9895497 | 0.9896436 |
| Lmean | Mountainous | 14.8773112 | 8.5765685 | 4.1278703 | 8.5157309 | 12.1435023 | 21.3542221 | 25.8825899 | 28.1982332 | 33.0075738 |
| Lmean | Overall | 15.4713025 | 9.0227921 | 4.1278703 | 8.8461982 | 13.0073201 | 19.5767712 | 26.6981627 | 32.4406946 | 40.1569670 |
| Lmean | Urban | 16.0652938 | 9.7120044 | 5.0305546 | 8.8999881 | 15.2159681 | 19.1816511 | 27.9325434 | 34.2705797 | 40.1569670 |
| REC | Mountainous | 0.0546851 | 0.0401839 | 0.0139477 | 0.0245139 | 0.0358738 | 0.0881934 | 0.1120347 | 0.1188076 | 0.1322695 |
| REC | Overall | 0.0660022 | 0.0517533 | 0.0093528 | 0.0299942 | 0.0462215 | 0.0930734 | 0.1168259 | 0.1676264 | 0.2210050 |
| REC | Urban | 0.0773193 | 0.0604891 | 0.0093528 | 0.0374769 | 0.0620824 | 0.0922541 | 0.1639769 | 0.2038899 | 0.2210050 |
| TREND | Mountainous | -0.0000297 | 0.0000899 | -0.0001841 | -0.0000468 | -0.0000267 | -0.0000074 | 0.0000747 | 0.0001275 | 0.0001469 |
| TREND | Overall | -0.0000567 | 0.0001024 | -0.0003904 | -0.0001196 | -0.0000277 | -0.0000051 | 0.0000148 | 0.0000992 | 0.0001469 |
| TREND | Urban | -0.0000836 | 0.0001099 | -0.0003904 | -0.0001230 | -0.0000744 | -0.0000072 | -0.0000016 | 0.0000216 | 0.0000748 |
| Vmean | Mountainous | 19.3023123 | 11.6660081 | 4.2902308 | 9.8797156 | 15.4990452 | 28.6679785 | 34.1724025 | 37.1460312 | 42.7875352 |
| Vmean | Overall | 19.8928357 | 11.9917177 | 4.2902308 | 10.2294178 | 17.1413577 | 26.2250307 | 35.1725854 | 41.1603916 | 51.4847762 |
| Vmean | Urban | 20.4833591 | 12.6898034 | 5.5055365 | 10.9216524 | 20.5527973 | 24.5827549 | 35.9043798 | 42.8655952 | 51.4847762 |

## Paired T test after log transformation for each variable for the 2 groups (alternative hypothesis: true difference in means is not equal to 0)

Paired - T test


| metric | estimate | statistic | p.value | parameter | conf.low | conf.high | method | alternative |
| --- | --- | --- | --- | --- | --- | --- | --- | --- |
| REC | 0.0204912 | 1.4229681 | 0.1766458 | 14 | -0.0103944 | 0.0513767 | Paired t-test | two.sided |
| DET | 0.0040894 | 0.4138454 | 0.6852574 | 14 | -0.0171043 | 0.0252832 | Paired t-test | two.sided |
| DIV | 0.0000221 | 0.0200802 | 0.9842628 | 14 | -0.0023421 | 0.0023864 | Paired t-test | two.sided |
| Lmean | 0.0687919 | 0.3487547 | 0.7324628 | 14 | -0.3542673 | 0.4918511 | Paired t-test | two.sided |
| ENTR | -0.0072113 | -0.1320289 | 0.8968402 | 14 | -0.1243574 | 0.1099349 | Paired t-test | two.sided |
| TREND | -0.0000539 | -2.1169674 | 0.0526522 | 14 | -0.0001084 | 0.0000007 | Paired t-test | two.sided |
| LAM | 0.0050832 | 0.4383629 | 0.6678117 | 14 | -0.0197874 | 0.0299538 | Paired t-test | two.sided |
| Vmean | 0.0596424 | 0.2675238 | 0.7929668 | 14 | -0.4185214 | 0.5378062 | Paired t-test | two.sided |
| Dim.1 | 0.0858369 | 0.4557899 | 0.6555307 | 14 | -0.3180813 | 0.4897551 | Paired t-test | two.sided |

## Leptin,cortisol and adiponectin are adjusted for creatinine and then log transformed (Metrics are log transformed in a previous step)

## Leptin models

##### Formula:leptin ~ Mesurment + SampleType + Age + (1 | ID) Number of observations:60

p.values values for Metabolites (14 total models)


| MesurmentName | effect | term | estimate | std.error | statistic | df | p.value | conf.low | conf.high |
| --- | --- | --- | --- | --- | --- | --- | --- | --- | --- |
| LAM | fixed | Mesurment | 0.2087057 | 0.1219579 | 1.7112928 | 55.92549 | 0.0925687 | -0.0356125 | 0.4530239 |
| TREND | fixed | Mesurment | -0.2571623 | 0.1509135 | -1.7040372 | 53.83889 | 0.0941399 | -0.5597464 | 0.0454219 |
| DET | fixed | Mesurment | 0.2105845 | 0.1262688 | 1.6677483 | 55.90719 | 0.1009582 | -0.0423715 | 0.4635406 |
| Dim.1 | fixed | Mesurment | 0.1336580 | 0.1226541 | 1.0897156 | 55.53002 | 0.2805449 | -0.1120935 | 0.3794096 |
| DIV | fixed | Mesurment | -0.1088072 | 0.1210671 | -0.8987340 | 55.34097 | 0.3726882 | -0.3513975 | 0.1337832 |
| REC | fixed | Mesurment | 0.1178744 | 0.1377325 | 0.8558207 | 55.66487 | 0.3957659 | -0.1580737 | 0.3938224 |
| ENTR | fixed | Mesurment | -0.0526780 | 0.1360764 | -0.3871207 | 55.80136 | 0.7001394 | -0.3252932 | 0.2199372 |
| Vmean | fixed | Mesurment | 0.0267850 | 0.1239815 | 0.2160404 | 54.75232 | 0.8297591 | -0.2217047 | 0.2752747 |
| Lmean | fixed | Mesurment | 0.0105629 | 0.1258745 | 0.0839164 | 55.01920 | 0.9334275 | -0.2416932 | 0.2628191 |

## Adiponectin models

##### Formula:adiponectin ~ Mesurment + SampleType + Age + (1 | ID) Number of observations:60

p.values values for Metabolites (14 total models)


| MesurmentName | effect | term | estimate | std.error | statistic | df | p.value | conf.low | conf.high |
| --- | --- | --- | --- | --- | --- | --- | --- | --- | --- |
| LAM | fixed | Mesurment | 0.2078839 | 0.1274885 | 1.6306086 | 54.93696 | 0.1086936 | -0.0476154 | 0.4633832 |
| DET | fixed | Mesurment | 0.2117652 | 0.1309851 | 1.6167123 | 53.08482 | 0.1118683 | -0.0509478 | 0.4744782 |
| Dim.1 | fixed | Mesurment | 0.1205603 | 0.1288743 | 0.9354876 | 55.84094 | 0.3535651 | -0.1376222 | 0.3787428 |
| DIV | fixed | Mesurment | -0.1103397 | 0.1270831 | -0.8682485 | 55.96513 | 0.3889664 | -0.3649212 | 0.1442418 |
| REC | fixed | Mesurment | 0.0640159 | 0.1433105 | 0.4466937 | 52.47101 | 0.6569345 | -0.2234962 | 0.3515280 |
| ENTR | fixed | Mesurment | 0.0605180 | 0.1404926 | 0.4307557 | 52.28497 | 0.6684166 | -0.2213645 | 0.3424004 |
| TREND | fixed | Mesurment | -0.0523245 | 0.1584376 | -0.3302534 | 48.21072 | 0.7426394 | -0.3708487 | 0.2661996 |
| Vmean | fixed | Mesurment | 0.0251200 | 0.1304386 | 0.1925808 | 55.90310 | 0.8479852 | -0.2361900 | 0.2864300 |
| Lmean | fixed | Mesurment | 0.0056902 | 0.1322686 | 0.0430202 | 55.96965 | 0.9658385 | -0.2592788 | 0.2706593 |

## Cortisol models

##### Formula:cortisol ~ Mesurment + SampleType + Age + (1 | ID) Number of observations:60

p.values values for Metabolites (14 total models)


| MesurmentName | effect | term | estimate | std.error | statistic | df | p.value | conf.low | conf.high |
| --- | --- | --- | --- | --- | --- | --- | --- | --- | --- |
| DIV | fixed | Mesurment | -0.1567154 | 0.1152423 | -1.3598775 | 46.51265 | 0.1804242 | -0.3886172 | 0.0751863 |
| Dim.1 | fixed | Mesurment | 0.1324428 | 0.1171310 | 1.1307236 | 45.56476 | 0.2640882 | -0.1033905 | 0.3682760 |
| Lmean | fixed | Mesurment | 0.1222050 | 0.1213800 | 1.0067971 | 49.01239 | 0.3189771 | -0.1217156 | 0.3661256 |
| Vmean | fixed | Mesurment | 0.1193946 | 0.1202125 | 0.9931960 | 49.87494 | 0.3254087 | -0.1220743 | 0.3608635 |
| REC | fixed | Mesurment | 0.1181171 | 0.1262031 | 0.9359284 | 38.41459 | 0.3551569 | -0.1372773 | 0.3735115 |
| ENTR | fixed | Mesurment | 0.1045907 | 0.1226674 | 0.8526370 | 36.60289 | 0.3994055 | -0.1440480 | 0.3532295 |
| LAM | fixed | Mesurment | 0.0991907 | 0.1169938 | 0.8478281 | 42.48367 | 0.4012852 | -0.1368329 | 0.3352142 |
| DET | fixed | Mesurment | 0.0946983 | 0.1186779 | 0.7979436 | 39.11713 | 0.4297209 | -0.1453275 | 0.3347241 |
| TREND | fixed | Mesurment | -0.0978420 | 0.1334524 | -0.7331601 | 29.58670 | 0.4692304 | -0.3705478 | 0.1748639 |

## Metrics ~ setting models

##### Formula:Mesurment ~ setting + Age + Sex Number of observations:62

| MesurmentName | term | estimate | std.error | statistic | p.value | conf.low | conf.high |
| --- | --- | --- | --- | --- | --- | --- | --- |
| TREND | settingMountainous | 0.5611994 | 0.2198518 | 2.5526260 | 0.0133460 | 0.1302978 | 0.9921010 |
| REC | settingMountainous | -0.4693247 | 0.2477765 | -1.8941453 | 0.0631958 | -0.9549576 | 0.0163083 |
| Dim.1 | settingMountainous | -0.1651980 | 0.2546411 | -0.6487484 | 0.5190616 | -0.6642854 | 0.3338894 |
| LAM | settingMountainous | -0.1495309 | 0.2566789 | -0.5825601 | 0.5624475 | -0.6526123 | 0.3535505 |
| DET | settingMountainous | -0.1293282 | 0.2547592 | -0.5076489 | 0.6136247 | -0.6286472 | 0.3699907 |
| Lmean | settingMountainous | -0.1267240 | 0.2587988 | -0.4896622 | 0.6262199 | -0.6339604 | 0.3805124 |
| Vmean | settingMountainous | -0.1032547 | 0.2598492 | -0.3973640 | 0.6925577 | -0.6125498 | 0.4060403 |
| ENTR | settingMountainous | 0.0594521 | 0.2567920 | 0.2315185 | 0.8177269 | -0.4438510 | 0.5627552 |
| DIV | settingMountainous | -0.0165348 | 0.2562612 | -0.0645232 | 0.9487755 | -0.5187976 | 0.4857280 |
